# Single-cell transcriptome sequencing analysis of peripheral blood mononuclear cells reveals immune signatures in patients with polymyalgia rheumatica

**DOI:** 10.3389/fimmu.2026.1852877

**Published:** 2026-10-19

**Authors:** Tiantian Cai, Qiu Qin, Yanfei Jiang, Zhou Liu, Ronghua Song, Jin-an Zhang

**Affiliations:** 1 Department of Endocrinology and Rheumatology, Shanghai University of Medicine and Health Sciences Affiliated Zhoupu Hospital, Shanghai, China; 2 Department of Endocrinology, The First Affiliated Hospital of Bengbu Medical University, Bengbu, China; 3 Department of Obstetrics and Gynecology, Shanghai University of Medicine and Health Sciences Affiliated Zhoupu Hospital, Shanghai, China

**Keywords:** cellular communication, immune signatures, peripheral blood mononuclear cells, polymyalgia rheumatica, single-cell transcriptome sequencing

## Abstract

**Background:**

Polymyalgia rheumatica (PMR) is an auto-inflammatory disease or autoimmune disease that involves multiple immune cells. In this study, single-cell transcriptome sequencing (scRNA-seq) combined with cellular communication analysis were used to investigate immune characteristics of peripheral blood mononuclear cells (PBMCs) in PMR patients.

**Methods:**

Three newly diagnosed PMR patients were recruited; their PBMCs were isolated and sequenced on the Illumina NovaSeq 6000 sequencing platform. The PBMCs scRNA-seq data of three normal controls were downloaded from the 10× Genomics platform for joint analysis.

**Results:**

Relative to healthy controls, PMR patients exhibited a reduced abundance of circulating T cells, alongside an expanded megakaryocyte/platelet compartment. At the T cell subset level, PMR was associated with an increased proportion of pro-inflammatory KIR^+^NK-like T cells, while CD4^+^ central memory T cells, effector memory T cells, and Th1 cells showed reductions in relative abundance. Circulating NK cell subsets were disproportionately represented in PMR patients; notably, the CD56^dim^CD16^hi^-ISG subset was exclusively detected in PMR cases. While no statistically meaningful shifts were observed in the global proportional distribution of B cells, the unswitched memory B cell compartment was absent in PMR patients. Multiple monocyte subsets annotated for elevated inflammatory and interferon-stimulated gene signatures, including CD14_Monocyte-ISG and CD16_Monocyte-IFITM1, were uniformly expanded in the PMR cohort. Functional enrichment analysis showed that the co-stimulation, cytotoxicity, antigen processing and presentation, and inflammation promotion of various immune cells in PMR patients were all enhanced. Key inflammatory pathways were widely activated, including the interferon-γ signaling pathway, interleukin-1β production, and NK cell-mediated cytotoxicity. Cellular communication analysis revealed more interactions but slightly weaker strength in PMRs, with increased communication in TGF-β and CD48 pathways.

**Conclusions:**

This preliminary study may help to further elucidate the pathogenesis of PMR and provide a theoretical basis for identifying diagnostic markers and discovering new therapeutic targets.

## 1 Introduction

Polymyalgia rheumatica (PMR) is a syndrome characterized by muscle pain and morning stiffness in the neck, scapular girdle and pelvic girdle and elevated erythrocyte sedimentation rate (ESR), with or without systemic reactions such as fever (1). Epidemiological studies have shown that the incidence of PMR varies according to age, gender, race, geography, and season (1, 2). It occurs mainly in people over 50 years of age, but less frequently in people under 50 years of age. Its incidence increases with age, with a peak age of onset around 70–80 years, and is 2-2.5 times higher in women than in men (1). PMR is more common in Northern Europeans and people of Nordic descent, but less common in Southern Europeans, Asians, Africans, African Americans, and Hispanics (2). There are no complete statistics data on the global incidence of PMR, and different studies have shown that the prevalence of PMR ranges from 0.37% to 1.53% (1–3). Although there are no epidemiological data on PMR in China, the disease is not uncommon in clinical practice.

The main clinical manifestations of PMR are muscle pain and stiffness in the trunk and proximal limbs of the extremities, with no specific laboratory test indicators or pathological changes, making it more of a diagnosis of exclusion (4). In people over 50 years of age with unexplained fever, increased ESR and moderate anemia and with difficulty in lifting arms, dressing, squatting and getting up, the diagnosis of PMR needs to be considered after exclusion of other diseases with clinical manifestations similar to those of PMR, such as rheumatoid arthritis (RA), inflammatory myopathies, hypothyroidism, Parkinson’s disease, neoplasms, and infections (1).

The pathogenesis of PMR remains unclear and is thought to be an auto-inflammatory disease or autoimmune disease that involves genetic susceptibility, environment, age, and abnormalities in the immune system (1–4). Recent systematic reviews, through in - depth analysis of the characteristic immune activation background in PMR, have preliminarily clarified that molecular and cellular evidence confirms the presence of a complex interactive regulatory network between the innate and adaptive immune systems during the pathogenesis of PMR. This disease seems to be located in the intermediate transitional position within the continuum of auto-inflammatory and autoimmune spectra, and can be defined as an immune-mediated inflammatory disease driven by a mixed mechanism under the combined influence of genetic and epigenetic backgrounds, immune senescence, and endocrine senescence (5). However, this immunological positioning of PMR has not been validated at the single-cell level. Therefore, in order to better resolve the pathogenesis of PMR, there is an urgent need to explore the molecular mechanisms of PMR at the single-cell level. The advent of single-cell sequencing technology has effectively addressed the limitations of traditional transcriptome analysis in cell type identification. The technology enables the generation of more comprehensive mRNA expression profiles, thereby facilitating the accurate identification of cell subpopulations. This, in turn, opens up new research avenues for investigating the immune tolerance disruption and the dysregulation of inflammatory pathways at the single-cell level (6). Single-cell transcriptome sequencing (scRNA-seq) has been widely used in autoimmune diseases, which can effectively analyze the cellular heterogeneity of immune-inflammation-associated diseases, identify pathogenic cell subpopulations, and provide important technical support for elucidating the molecular mechanisms of disease development (7).

Therefore, in this study, we applied scRNA-seq combined with cellular communication in order to characterize the immune signatures of peripheral blood mononuclear cells (PBMCs) in PMR patients.

## 2 Materials and methods

### 2.1 Specimen collection

A total of three PMR patients were recruited from Department of Endocrinology & Rheumatology, Shanghai University of Medicine & Health Sciences Affiliated Zhoupu Hospital, according to the 2012 ACR/EULAR PMR classification criteria (8). These patients were newly diagnosed and not treated with any glucocorticoids, nonsteroidal anti-inflammatory drugs (NSAIDs), immunosuppressants or biologics. At the same time, we downloaded the datasets of scRNA-seq or the PBMCs of three normal controls for joint analysis from the 10×Genomics platform (https://www.10xgenomics.com/). All the subjects’ information is detailed in **Supplementary Table S1**. Written informed consent was obtained from all participants in accordance with the Declaration of Helsinki. The study was reviewed and approved by the Ethics Committee of Shanghai University of Medicine & Health Sciences (Approval number: 2023-C-123-E01).

### 2.2 PBMCs isolation, 10× library preparation and sequencing

PBMCs from all the patients were isolated and purified using density gradient sedimentation. Cells that passed the assay were resuspended to a suitable cell concentration of 700–1200 cells/μL, ready for manipulation on a 10× Genomics Chromium System. In this study, high-throughput gene expression profiling was performed at the single-cell level using the 10× Genomics Chromium System™, with the core technology being the use of microfluidic chips to accurately differentiate between cells, so that only one cell was present in each small reaction system, that is, Gel Bead in Emulsion (GEMs). The library construction was performed according to the standard manufacturer’s protocol (Chromium Single Cell 5′ v3.1). Sequencing libraries were quantified using a high sensitivity DNA chip (Agilent) on a Bioanalyzer 2100 and the Qubit High Sensitivity DNA Assay (Thermo Fisher Scientific). The libraries were sequenced on NovaSeq6000 (Illumina).

### 2.3 ScRNA-seq data processing

The raw image data obtained from Illumina sequencing were converted into sequence data (FASTQ format) by base calling, thus yielding the most original sequencing data files. Furthermore, we performed quality control of the raw data using the official 10× Genomics software, Cell Ranger (v7.1.0), which allowed us to use the alignment software STAR to align the reads obtained from sequencing to the human genome (build GRCh38), and to obtain cell number, gene number, and genome alignment information for the sample data. Cell Ranger is capable of distinguishing cells based on barcode sequence markers and quantifying single-cell transcriptome data using unique molecular identifier (UMI) markers for different mRNAs in each cell. Following the creation of an expression matrix by the Cell Ranger, the Seurat (v4.1.1) R tool kit was utilized as a subsequent quality control filter to eliminate outliers or exclude low-quality cells (such as doublets, dead cells, and cell debris). We filtered the matrices using a standard panel of three quality criteria: (1) the number of genes; (2) the number of UMIs; (3) mitochondrial and ribosomal genes expressed in each cell (Quartile threshold screening criteria). The expression of mitochondria genes and ribosomal genes was calculated using PercentageFeatureSet function of the Seurat package. Then we performed principal component analysis (PCA) and reduced the data to the top 30 PCA components after scaled the data. We visualized the clusters on a 2D map produced with t-distributed stochastic neighbor embedding (t-SNE) and uniform manifold approximation and projection (UMAP). To eliminate potential batch effects between the PMR and controls, we integrated data from different samples after identifying ‘anchors’ between datasets using FindIntegrationAnchors and IntegrateData in the Seurat package.

### 2.4 Marker gene identification and cell type annotation

In this study, differential expressed genes (DEGs) were identified as genes with |log2FC|>0.25 and Q value ≤ 0.05. The function FindMarkers in Seurat identified DEGs in each cluster by using the default algorithm Wilcoxon rank-sum test, which provides the basis for further screening of marker genes. For each cluster, we used the Wilcoxon rank-sum test to find DEGs by comparing the remaining clusters. To reduce the technical bias caused by small-sample data and lower the false discovery rate, DEGs analysis between PMRs and controls was performed using a pseudobulk approach using the bulk RNA-Seq tool DESeq2. Cell type annotation was performed using the SingleR package, based on the public single-cell reference expression quantification public dataset in combination with the published related literature.

### 2.5 Functional enrichment analysis

Functional enrichment analysis between groups was performed on those DEGs for each cell subtype using ClusterProfiler in R, Gene Ontology (GO) and Kyoto Encyclopedia of Genes and Genomes (KEGG) database (9, 10). To measure the immunoreactivity of different immunocytes subsets between PMRs and controls, we included the MsigDB database collection and literature reports of co-stimulatory molecules, cytotoxic molecules, antigen-presenting molecules, and pro-inflammatory cytokine gene sets for scoring and comparison (**Supplementary Table S2**) (11, 12). We also used gene set variation analysis (GSVA), with the background gene set file “h.all.v2024.1.Hs.symbols.gmt” downloaded from the MSigDB database. Then, individual cells were scored for pathway activity values using the GSVA package (v1.30.0). Finally, the LIMMA package (v3.38.3) was used to calculate the differences between different subgroups of signaling pathway activity.

### 2.6 Cell communication analysis

The CellChat platform (13) was used to systematically analyze the possible cellular interactions between subpopulations of PBMCs. Specifically, a cellular communication network was constructed based on scRNA-seq Seurat object in R, integrating the CellChat Shiny application. Default parameter settings were maintained during the analysis, focusing on communication mechanisms such as secreted signaling, ECM-receptor, and cell-cell contact relationships.

### 2.7 Statistical analysis

DEGs analysis was performed using the Wilcoxon rank-sum test or DESeq2. Statistical analysis of cell proportions was performed using the Wilcoxon rank-sum test and independent samples t-test in GraphPad Prism project, according to the characteristics of the data format. The *p-*values with statistical significance are indicated by an asterisk: *p < 0.05, **p < 0.01, and ***p < 0.001. However, given this small sample size, these statistical tests of proportions have extremely limited statistical power at the conventional α=0.05 threshold, and no formal statistical significance claims are made for these exploratory comparisons. All reported inter-group differences are presented as suggestive, hypothesis-generating observations, rather than robust validated associations.

## 3 Results

### 3.1 Single-cell transcriptome atlas of PBMCs

We first performed an overall analysis of single-cell transcriptome data from PMR patients and controls to characterize the single-cell profiles of PBMCs. We briefly summarized and evaluated the data, and after removing low-quality cells using quality control indicators, such as mitochondrial gene expression, median gene number, and valid UMIs, a total of 59,364 high-quality single-cell gene expression data (including 30,286 of controls and 29,078 of PMRs) were screened. The median reads per cell, median genes per cell, total genes detected and median UMI counts per cell for each sample are summarized in **Supplementary Table S3**. We identified ten clusters using UMAP unsupervised clustering based on each cellular gene expression profile, combining the SingleR package and known typical cell marker genes. These cells were annotated as T cells (CD3D, CD3E and CD3G), B cells (CD79A, CD79B and MS4A1), natural killer (NK) cells (NCAM1, NKG7 and KLRF1), CD14^+^monocytes (CD14, LYZ and CD68), CD16^+^monocytes (FCGR3A and CD68), granulocytes (CSF3R), plasmacytoid dendritic (pDC) cells (LILRA4, TCF4, FCER1A and CST3), megakaryocyte/platelets (PPBP and PF4), hemopoietic stem (HSCs) cells (CYTL1 and GATA2) and others/unknown (**Figures 1A, B**). The typical signature genes were specifically highly expressed in each of the major cell types, all of which have distinct gene expression patterns (**Figures 1C, D**). Compared to other cell types, other/unknown cells are deficient in expression of typical markers and highly express genes related to oxidative phosphorylation and mRNA metabolic process. In addition, a reduction of T cells and expansion of megakaryocyte/platelets were observed in PMRs based on the statistical analysis of cell abundance. CD14^+^monocytes were elevated in the PMRs at a marginal *p-*value. The proportions of other major cell types showed trends of elevation and decline, but did not reach statistical differences (**Figures 1E, F**; **Supplementary Table S4**).

**Figure 1:**
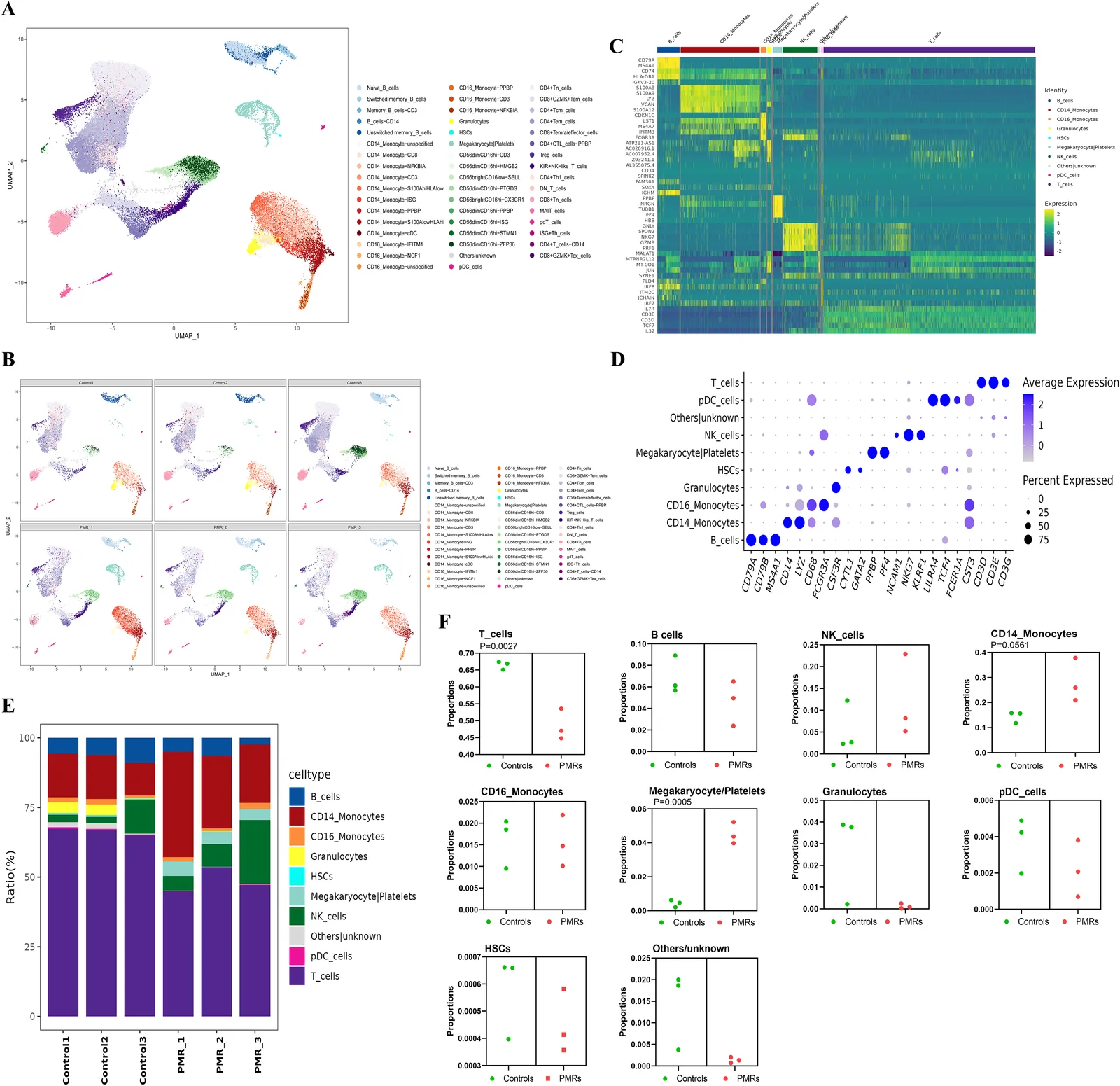
Identification of 10 PBMCs cell subsets. **(A)** UMAP embeddings and subset annotations of scRNA-Seq dataset from patients with PMRs and controls. **(B)** UMAP embedding for cell subsets in each sample. **(C)** Normalized expression of the top 50 ranked genes in different cell subsets. **(D)** Dot plots of marker genes in each cell subset. **(E)** Stacked bar chart showing the proportions of different cell types in each sample. **(F)** Statistical plots showing the proportions of cell subsets in patients and controls.

### 3.2 Single-cell transcriptome atlas of T, B and NK cells subpopulations

Based on the expression of marker genes in each subcluster (**Supplementary Table S5**), we defined T cells subpopulations as CD4^+^Tn_cells (naïve CD4^+^T cells, Cluster 0), CD4^+^Tcm_cells (central memory CD4^+^T cells, Cluster 1), CD4^+^Tem_cells (effector memory CD4^+^T cells, Cluster 2), CD8^+^GZMK^+^Tem_cells (GZMK^+^effector memory CD8^+^T cells, Cluster 3), CD8^+^Tn_cells (naïve CD8^+^T cells, Cluster 4), CD8^+^Temra/effector_cells (terminally differentiated effector memory/effector CD8^+^T cells, Cluster 5), KIR^+^NK-like T_cells (KIR^+^NKT cells, Cluster 6), CD4^+^Th1_cells (helper 1 CD4^+^T cells, Cluster 7), DN T_cells (double negative T cells, Cluster 8), Treg_cells (regulatory T cells, Cluster 9), CD4^+^CTL_cells-PPBP (CD4^+^ cytotoxic T lymphocytes, Cluster 10), MAIT_cells (mucosal-associated invariant T cells, Cluster 11), γδT_cells (gammadelta T cells, Cluster 12), CD4^+^T_cells-CD14 (Cluster 13), ISG^+^Th_cells (interferon-stimulated gene positive helper T cells, Cluster 14) and CD8^+^GZMK^+^Tex_cells (GZMK^+^exhausted CD8^+^T, Cluster 15) (**Figure 2A**). The average expression of the top 3 ranked genes in different T cell subsets is shown in **Figure 2B**. We then compared the differences in the proportions of T cells subpopulations between controls and PMRs. Among them, the proportions of CD4^+^Tcm cells, CD4^+^Tem cells and CD4^+^Th1 cells were reduced (*p* < 0.05 or marginal *p*-value) while the proportion of KIR^+^NK-like T cells was elevated in PMRs (*p* < 0.05, **Supplementary Table S4**; **Figure 2C**). KIR^+^NK-like T cells express the killer cell immunoglobulin-like receptor (KIR) family (KIR3DL1, KIR3DL2, KIR2DL3 and KIR2DL1) and killer cell lectin-like receptor (KLR) family (KLRC2, KLRD1, KLRF1, KLRK1, KLRC3, KLRC4, KLRG1 and KLRB1) in addition to cytotoxic genes (GNLY, NKG7, GZMB, PRF1, GZMH and GZMA). The proportions of the other T cells subtypes were not significantly different.

**Figure 2:**
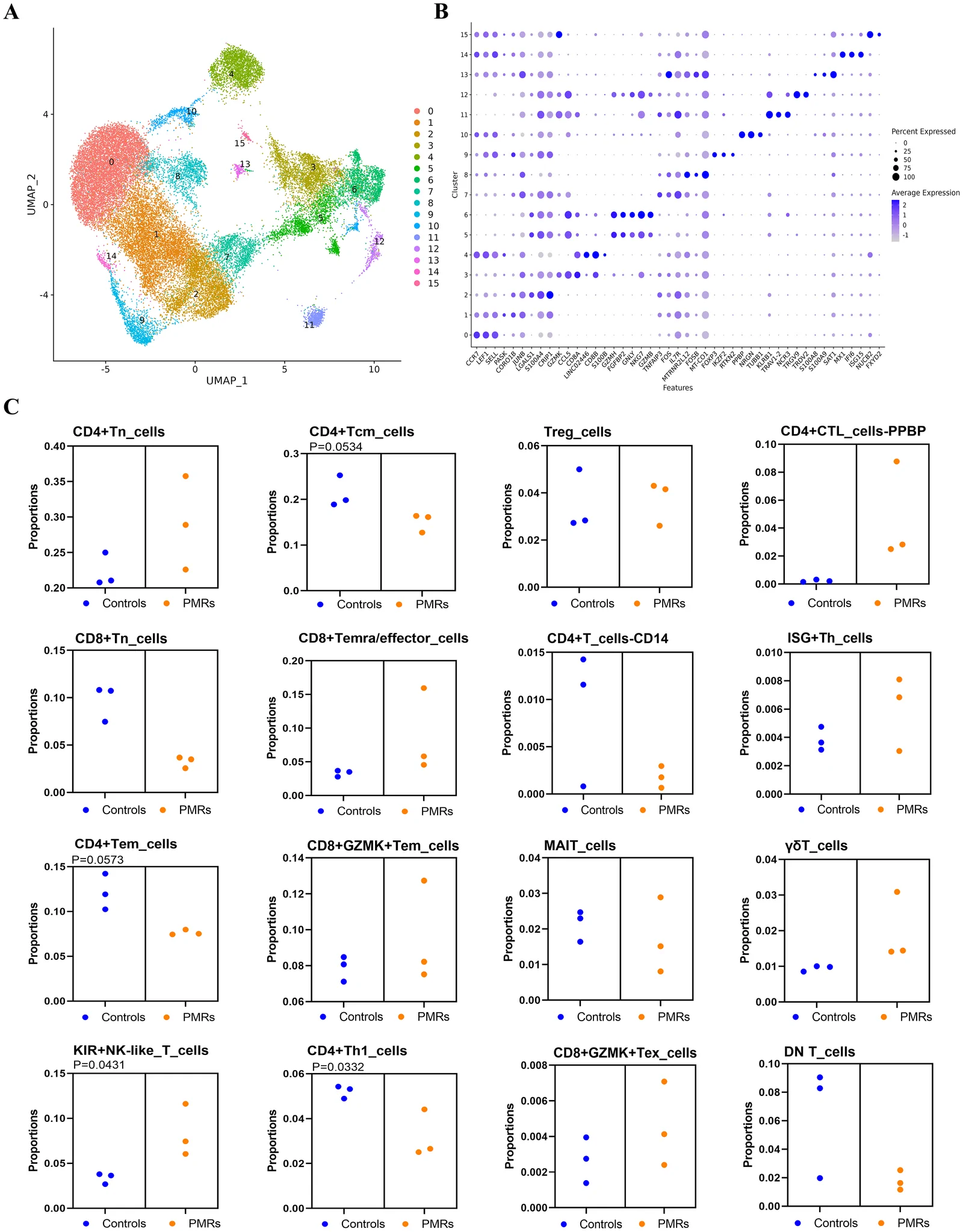
Single-cell transcriptome atlas of T cells subpopulations. **(A)** UMAP visualization of T cell clusters from patients with PMRs and controls. **(B)** Normalized expression of the top 3 ranked genes in different T cell subsets. **(C)** Statistical plots showing the proportions of T cell subsets in patients and controls.

In our study, we also defined B cells clusters as naive_B_cells (Cluster 0, 2 and 7), unswitched memory_B_cells (Cluster 1), switched memory_B_cells (Cluster 3 and 4), memory_B_cells-CD3 (Cluster 5) and B_cells-CD14 (Cluster 6) (**Figure 3A**; **Supplementary Table S5**). The average expression of the top 3 ranked genes in different B cell subsets is shown in **Figure 3B**. Naive B cells highly expressed IGHD, TCL1A and FCER2 molecules. Unswitched memory B cells retained IGHD expression in addition to CD27, while switched memory B cells highly expressed CD27, AIM2, TNFRSF13B, JCHAIN, IGHA1, IGHA2, IGHG1 and IGHG2. Memory B cells-CD3 expressed CD3D, CD3E and CD3G in addition to CD27 molecules, while B cells-CD14 expressed some of the characteristic markers of CD14 monocytes such as CD14, LYZ, S100A8, S100A9 and TYROBP. The proportions of these B cells subpopulations differed non-significantly between controls and PMRs, but unswitched memory B cells were not present in any of the PMR patients (**Supplementary Table S4**; **Figure 3C**).

**Figure 3:**
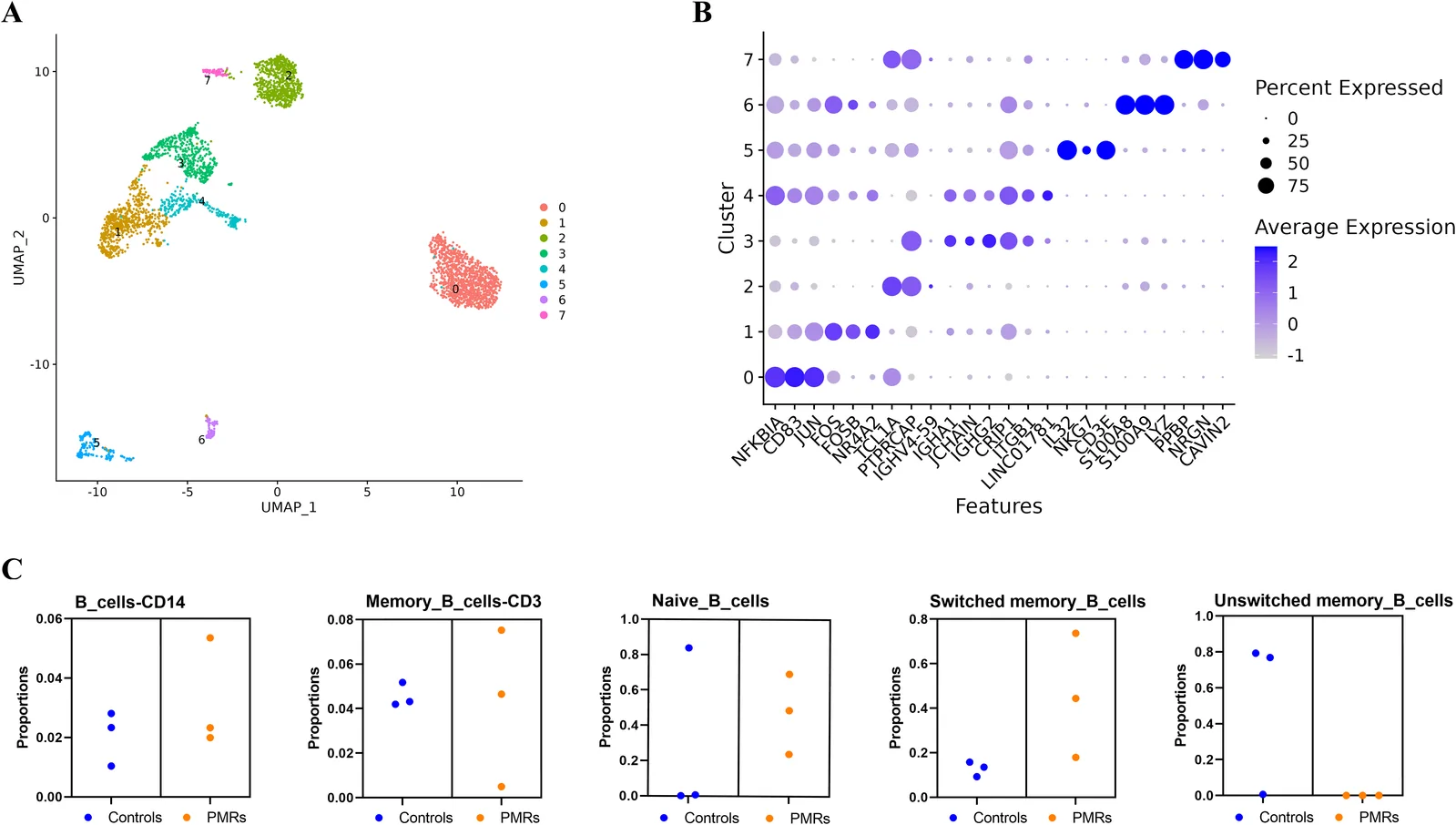
Single-cell transcriptome atlas of B cells subpopulations. **(A)** UMAP visualization of B cell clusters from patients with PMRs and controls. **(B)** Normalized expression of the top 3 ranked genes in different B cell subsets. **(C)** Statistical plots showing the proportions of B cell subsets in patients and controls.

For NK cells, we first classified them into CD56^dim^CD16^hi^ as well as CD56^bright^CD16^low^ subpopulations based on the expression of NCAM1 (CD56) and FCGR3A (CD16), and we also identified one transitional subpopulation CD56^bright^CD16^hi^ (**Figure 4A**). Then, based on the expression of marker genes in each subcluster, we defined these NK cells subpopulations as CD56^dim^CD16^hi^-PTGDS (Cluster 0), CD56^dim^CD16^hi^-ZFP36 (Cluster 1), CD56^bright^CD16^hi^-CX3CR1(Cluster 2), CD56^bright^CD16^low^-SELL (Cluster 3), CD56^dim^CD16^hi^-CD3 (Cluster 4), CD56^dim^CD16^hi^-PPBP (Cluster 5), CD56^dim^CD16^hi^-STMN1 (Cluster 6), CD56^dim^CD16^hi^-ISG (Cluster 7) and CD56^dim^CD16^hi^-HMGB2 (Cluster 8)(**Supplementary Table S5**; **Figure 4B**). The average expression of the top 10 ranked genes in different NK cell subsets is shown in **Figure 4C**. Compared with other clusters, the CD56^dim^CD16^hi^-PTGDS cells, in addition to high expression of PTGDS, also expressed the chemokine receptors (CX3CR1 and CXCR2), the cytotoxic molecules (GZMH, GZMA, GZMB and PRF1), as well as the inflammatory molecules (CCL4L2, CCL5, and CCL4). The CD56^dim^CD16^hi^-ZFP36 cells predominantly expressed cellular stress-related molecules (ZFP36, FOS, JUNB, DUSP1, NR4A2, DUSP2, JUN, NFKBIA, RGS2 and DNAJB1). The CD56^bright^CD16^hi^-CX3CR1 transitional cells expressed both CD56^bright^ and CD56^dim^ subpopulation related molecules such as IL2RB, CX3CR1, CD160 and KLRD1. CD56^bright^CD16^low^-SELL cells highly expressed the signature of NK cell precursors including SELL and IL7R, and were mapped at the early developmental stage. CD56^dim^CD16^hi^-CD3 cells expressed some characteristic genes of naïve T cells such as IL7R, LTB, TCF7, CCR7, LEF1, SELL, CD3D, CD3E and CD3G. CD56^dim^CD16^hi^-PPBP cells highly expressed the platelet signature genes PPBP and PF4. CD56^dim^CD16^hi^-STMN1 and CD56^dim^CD16^hi^-HMGB2 cells expressed cell proliferation-related molecules such as STMN1, HMGB2 and MKI67. CD56^dim^CD16^hi^-ISG cells expressed interferon-stimulated genes (ISGs) like MX1, OAS1, IFI44L, ISG15, XAF1, IFIT3, IFI6, IRF7, OAS2 and STAT1. The proportions of CD56^dim^CD16^hi^-PTGDS and CD56^dim^CD16^hi^-PPBP were higher in patients with PMR (*p*-value <0.01 and <0.0001, respectively). Specially, CD56^dim^CD16^hi^-ISG was only present in PMR patients (**Supplementary Table S4**; **Figure 4D**).

**Figure 4:**
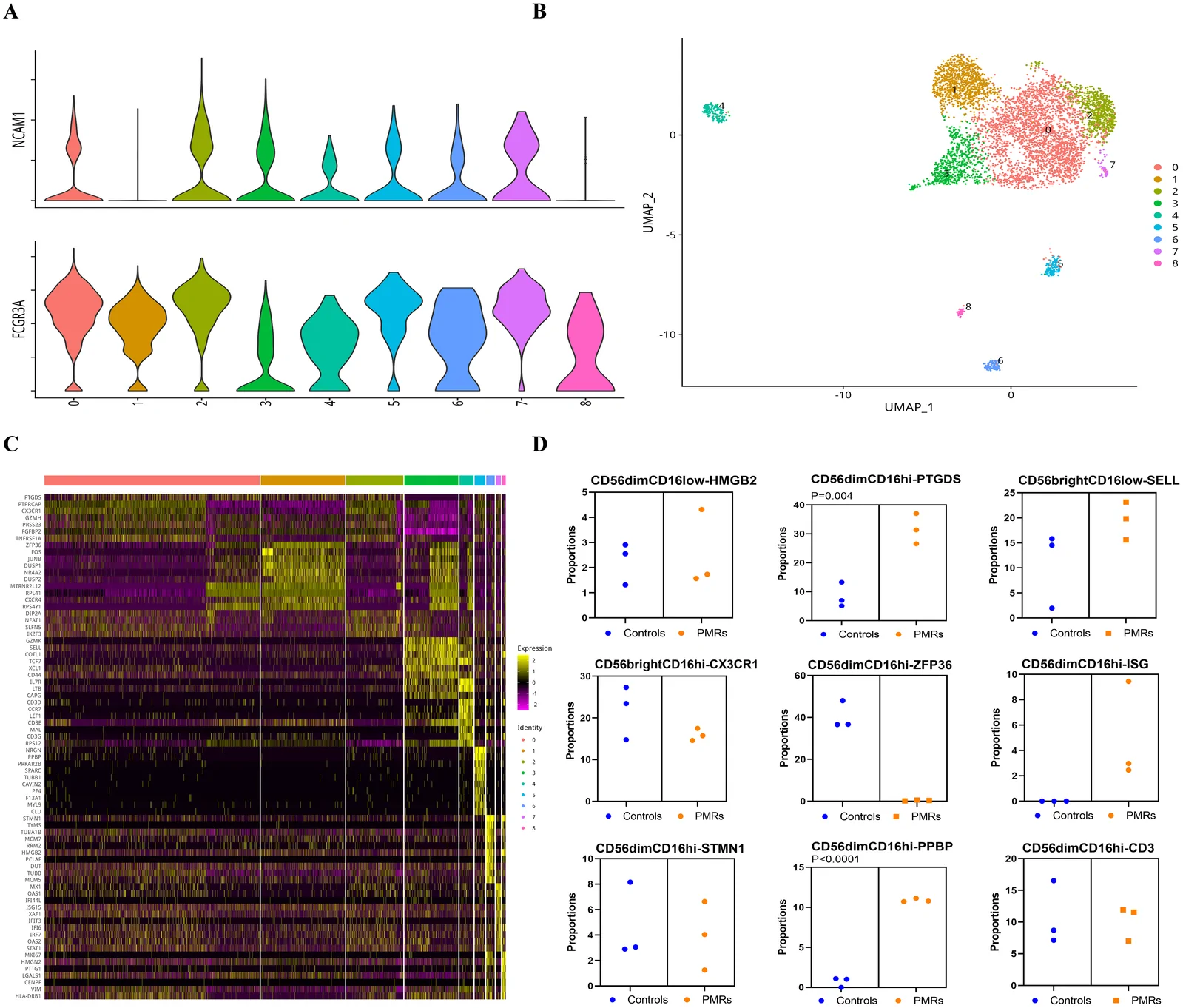
Single-cell transcriptome atlas of NK cells subpopulations. **(A)** Violin plots of NCAM1 and FCGR3A gene expression in each cluster. **(B)** UMAP visualization of NK cell clusters from patients with PMRs and controls. **(C)** Normalized expression of the top 10 ranked genes in different NK cell subsets. **(D)** Statistical plots showing the proportions of NK cell subsets in patients and controls.

### 3.3 Single-cell transcriptome atlas of monocytes subpopulations

In our study, monocytes were first classified into CD14^+^monocytes (CD14, LYZ and CD68), and CD16^+^monocytes (FCGR3A and CD68). Then, based on the expression of marker genes in each subcluster (**Supplementary Table S5**), we defined these subpopulations as CD14_Monocyte-NFKBIA (Cluster 0), CD14_Monocyte-unspecified (Cluster 1), CD14_Monocyte-S100A^hi^HLA^low^ (Cluster 2), CD14_Monocyte-S100A^low^HLA^hi^ (Cluster 3), CD14_Monocyte-ISG (Cluster 4), CD14_Monocyte-PPBP (Cluster 5), CD14_Monocyte-CD3 (Cluster 6), CD14_Monocyte-CD8 (Cluster 7) and CD14_Monocyte-cDC (Cluster 8) (**Figure 5A**), CD16_Monocyte-NCF1 (Cluster 0), CD16_Monocyte-IFITM1 (Cluster 1), CD16_Monocyte-NFKBIA (Cluster 2), CD16_Monocyte-unspecified (Cluster 3), CD16_Monocyte-CD3 (Cluster 4) and CD16_Monocyte-PPBP (Cluster 5) (**Figure 5C**). The average expression of the top 10 ranked genes in different CD14^+^ and CD16^+^ monocytes subsets is shown in **Figures 5B, D**. CD14_Monocyte-NFKBIA cells highly expressed cellular stress-related genes (NFKBIA, NFKBIZ, FOSB, JUN, NR4A2, FOS, PLAUR and PPP1R15A) and inflammatory genes (IL1B, CXCL8 and CCL3). CD14_Monocyte-unspecified cells lack typical markers. CD14_Monocyte-S100A^hi^HLA^low^ cells highly expressed the S100A gene family (S100A8, S100A12, S100A6, S100A4 and S100A11) but lowly expressed the HLA gene family, while CD14_Monocyte-S100A^low^HLA^hi^ cells highly expressed the HLA gene family (HLA-DPB1, HLA-DPA1, HLA-DRA, HLA-DRB1, HLA-DQA1, HLA-DMA, HLA-DRB5, HLA-DQB1, HLA-DMB and HLA-DQA2) but lowly expressed the S100A gene family. CD14_Monocyte-ISG cells expressed ISGs like MX1, ISG15, LY6E, IFI44L, XAF1, IFI6, OAS2, IFIT2, IFITM3 and OAS3. CD14_Monocyte-PPBP cells expressed platelet-related genes PPBP and PF4. CD14_Monocyte-CD3 cells expressed characteristic genes of naïve T cells such as IL7R, LTB, TCF7, CCR7, LEF1, SELL, CD3D, CD3E and CD3G. CD14_Monocyte-CD8 cells expressed cytotoxic (NKG7, GNLY, PRF1, CST7, CTSW, GZMA, GZMB, GZMH, GZMM and GZMK) and inflammatory (CCL5 and IL32) molecules in addition to CD8 molecules (CD8A and CD8B). CD14_Monocyte-cDC cells expressed marker genes of conventional dendritic cells (CLEC10A, FCER1A and CD1C) and also highly expressed HLA genes (HLA-DQA1, HLA-DPB1, HLA-DQB1, HLA-DPA1, HLA-DRA, HLA-DRB1, HLA-DRB5, HLA-DMA, HLA-DQA2, HLA-DMB, HLA-DOA and HLA-DQB2). CD16_Monocyte-NCF1 cells, in addition to expressing NCF1, also expressed some of the characteristic genes of monocyte (S100A8, MS4A6A, LYZ and S100A9). CD16_Monocyte-IFITM1 cells highly expressed ISGs like IFITM1, OAS1, IFITM2 and SAMD9. CD16_Monocyte-NFKBIA cells highly expressed stress genes (FOS, JUN, FOSB, NR4A2, ZFP36, DUSP1, NFKBIA and NFKBIZ) and inflammatory genes (CXCL16, CXCL8 and L1B). CD16_Monocyte-unspecified cells lacked typical markers. CD16_Monocyte-CD3 cells expressed characteristic genes of T cells. CD16_Monocyte-PPBP cells expressed platelet-related genes PPBP and PF4. The proportions of CD14_Monocyte-ISG cells, CD16_Monocyte-IFITM1 cells and CD16_Monocyte-NCF1 cells were higher in patients with PMR (*p*-value <0.05 and <0.01, respectively), while the proportion of CD16_Monocyte-NFKBIA cells was reduced (*p*-value <0.0001; **Supplementary Table S4** and **Figure 5E**).

**Figure 5:**
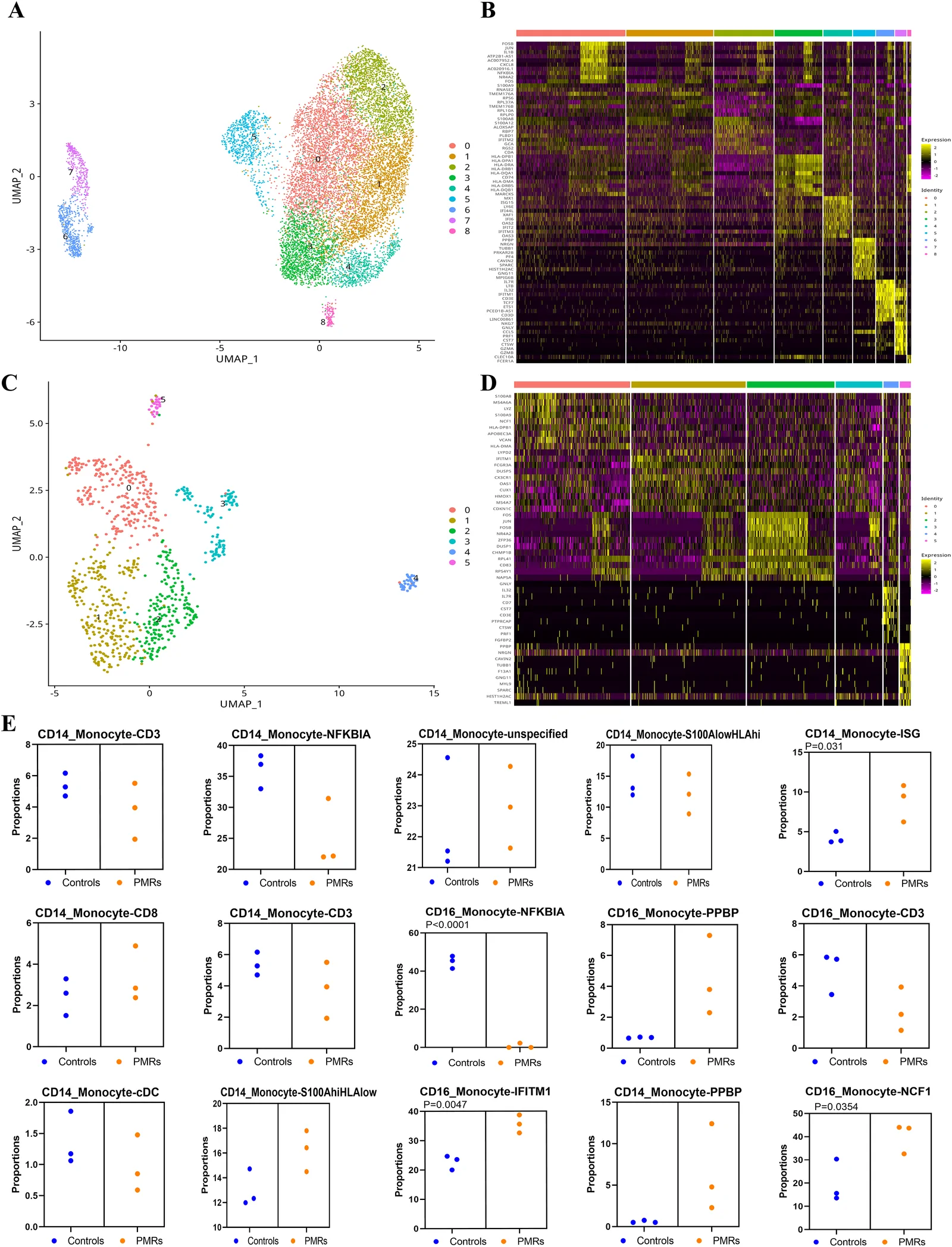
Single-cell transcriptome atlas of monocytes subpopulations. **(A)** UMAP visualization of CD14^+^monocyte clusters from patients with PMRs and controls. **(B)** Normalized expression of the top 10 ranked genes in different CD14^+^monocyte subsets. **(C)** UMAP visualization of CD16^+^monocyte clusters from patients with PMRs and controls. **(D)** Normalized expression of the top 10 ranked genes in different CD16^+^monocyte subsets. **(E)** Statistical plots showing the proportions of CD14^+^ and CD16^+^monocyte subsets in patients and controls.

### 3.4 Functional enrichment analysis

We compared the transcriptomes of PBMCs (1,051 up-regulated and 335 down-regulated), T cells (849 up-regulated and 230 down-regulated), B cells (670 up-regulated and 284 down-regulated), NK cells (1,115 up-regulated and 472 down-regulated), CD14^+^monocytes (738 up-regulated and 510 down-regulated) and CD16^+^monocytes (275 up-regulated and 321 down-regulated) in the controls and PMRs (**Supplementary Table S6**). We further analyzed these DEGs for functional enrichment. In comparison with the control group, the PMRs group clearly showed immune activation states. In terms of biological process (GO items), PBMCs in the PMRs exhibited leukocyte aggregation, interleukin-1β production, natural killer cell-mediated immunity, and leukocyte activation involved in the inflammatory response (**Figure 6A**). The biological processes involved in T cells activation in PMRs were negative regulation of interleukin-2 production, positive regulation of T cell migration, regulation of T cell receptor signaling pathway, regulation of T cell co-stimulation and regulation of T cell mediated cytotoxicity (**Figure 6B**). B cells in the PMRs group showed some biological processes of B cell activation such as B cell activation involved in immune response, regulation of B cell proliferation and B cell differentiation (**Figure 6C**). The biological processes activated in NK cells then involved regulation of lymphocyte chemotaxis, interferon-gamma production, positive regulation of granzyme B production and regulation of natural killer cell mediated cytotoxicity (**Figure 6D**). Meanwhile, for CD14^+^monocytes and CD16^+^monocytes, the biological processes activated in PMRs included interferon-gamma-mediated signaling pathway, regulation of interleukin-8 production, antigen processing, presentation of exogenous peptide antigen and positive regulation of mononuclear cell migration (**Figures 6E, F**). KEGG analysis of T cells showed significant activation in T-cell function in the PMRs, such as Th17 cell differentiation and Th1 and Th2 cell differentiation, suggesting that CD4^+^ T cell subsets were involved in the pathogenesis of PMRs (**Figure 6H**). We also found that the following pathways such as natural killer cell mediated cytotoxicity, chemokine signaling pathway, platelet activation, and NOD-like receptor signaling pathway were activated in most of the cell types. Also, KEGG analysis revealed evidence of pathogen infection, like human cytomegalovirus and salmonella infection (**Figures 6G–L**). The GSVA results further demonstrated the activated pathways such as apical junction, complement, interferon alpha response, interferon gamma response and glycolysis in PMRs. (**Supplementary Figure S1A**). Cellular immunoreactivity scores showed increased antigen processing and presentation by B cells, CD14^+^monocytes, CD16^+^monocytes and pDC in PMR group. Meanwhile, the expression of cytotoxic molecules was increased in CD14^+^monocytes, CD16^+^ monocytes, NK cells, and T cells, suggesting that their cytotoxicity in PMRs were enhanced. In addition, the expression of co-stimulatory molecules of CD14^+^ monocytes, CD16^+^monocytes, NK cells, and T cells was increased in the PMR group, suggesting the role of these cells in T cell activation of PMRs. Finally, we found that B cells, monocytes (CD14^+^ and CD16^+^), NK cells, pDC, and T cells proinflammatory scores were also elevated, suggesting a role of these cells in the PMR inflammatory response (**Supplementary Figure S1B**). Moreover, the scoring of subpopulations of T cells, B cells, NK cells and monocytes is also shown in **Supplementary Figure S1C**-**S1G**, and between the two groups, these cell subpopulations were also altered to different degrees in terms of co-stimulation, cytotoxicity, antigen processing and presentation, and inflammation promotion.

**Figure 6:**
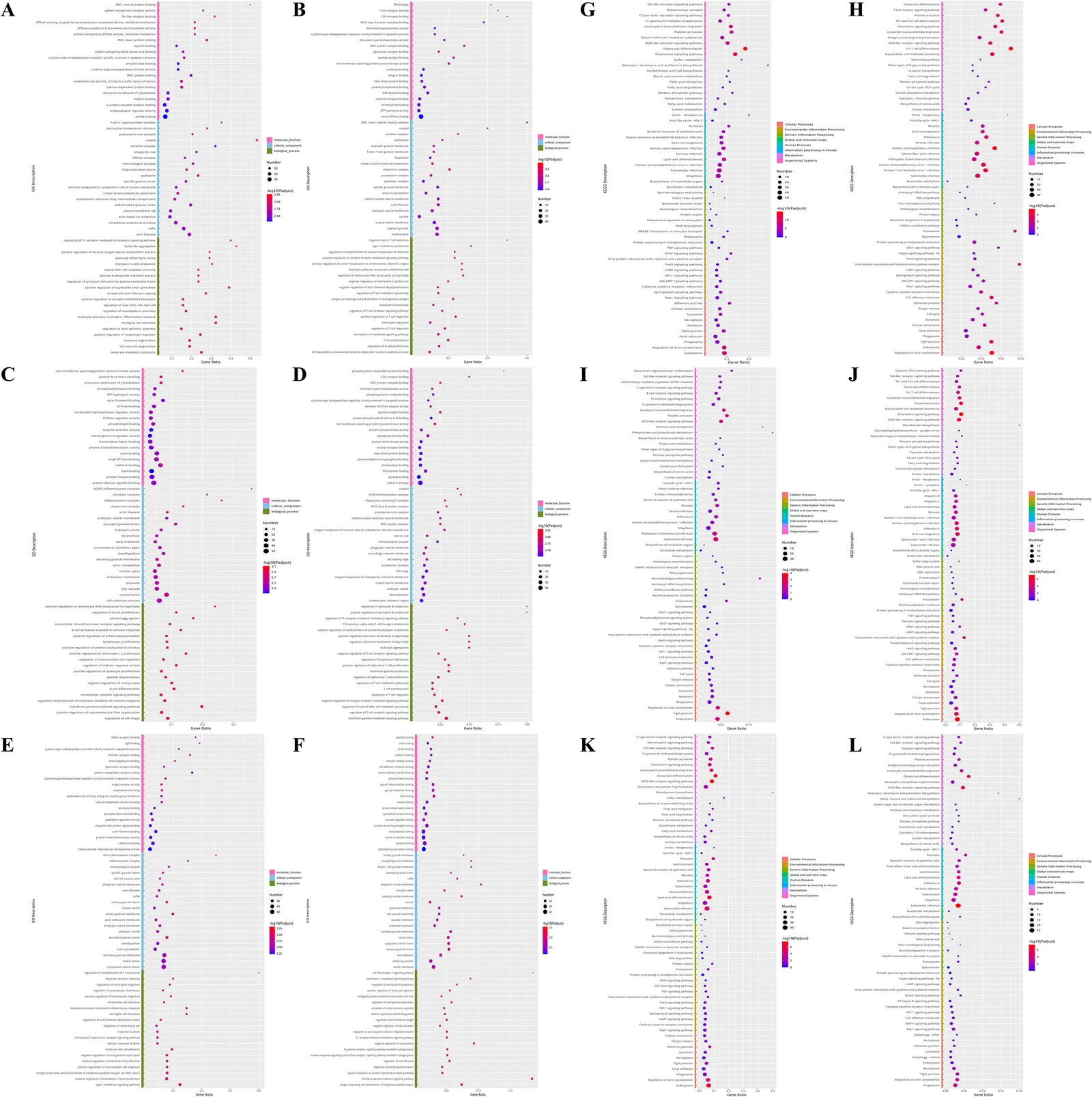
Functional enrichment analysis. **(A)** GO analysis of PBMCs DEGs from PMRs and controls. **(B)** GO analysis of T cells DEGs from PMRs and controls. **(C)** GO analysis of B cells DEGs from PMRs and controls. **(D)** GO analysis of NK cells DEGs from PMRs and controls. **(E)** GO analysis of CD14^+^ monocytes DEGs from PMRs and controls. **(F)** GO analysis of CD16^+^ monocytes DEGs from PMRs and controls. **(G)** KEGG analysis of PBMCs DEGs from PMRs and controls. **(H)** KEGG analysis of T cells DEGs from PMRs and controls. **(I)** KEGG analysis of B cells DEGs from PMRs and controls. **(J)** KEGG analysis of NK cells DEGs from PMRs and controls. **(K)** KEGG analysis of CD14^+^ monocytes DEGs from PMRs and controls. **(L)** KEGG analysis of CD16^+^ monocytes DEGs from PMRs and controls.

### 3.5 Cell communication analysis

The study of intercellular communication mechanisms can reveal the regulatory networks between different cell populations through ligand binding and signaling. Since cell differentiation processes and pathological states are often accompanied by specific communication patterns, elucidating these intercellular interactions is of great biological significance. In our study, cellular communication analysis between T subtypes, B subtypes, CD56^dim^NK cells, CD56^bright^NK cells, CD14^+^monocytes, CD16^+^monocytes, megakaryocyte/platelets, granulocytes, pDC cells and HSCs were performed.

To understand the differences in cellular communication between PBMCs from controls and PMRs, we first compared the number and strength of communication. Overall, the PMR group showed an increase in the number of cellular interactions, but a slight decrease in the interaction strength. Contrasting heatmaps of the number and strength of cellular interactions showed that the main cell populations involved in differential numbers of interactions (**Figures 7A–C**). Among them, KIR^+^NK-like T_cells and γδT_cells, as receptor cells, showed an increase in the number of communications with most of the ligand cells. Memory B cells-CD3, naive_B_cells, switched memory_B_cells and pDCs acted as ligand cells and all had an increased number of interactions with CD8^+^Temra/effector_cells. In contrast, MAIT_cells, as receptor cells, had a reduced number of communications with most ligand cells. The KIR^+^NK-like T_cells, CD8^+^Temra/effector_cells and MAIT_cells acted as receptor cells with reduced communication strength with most ligand cells (**Figure 7D**). Analysis of cell communication roles showed a decrease in incoming messages and an increase in outgoing messages for KIR^+^NK-like T_cells; a decrease in incoming messages and no significant change in outgoing messages for CD8+Temra/effector_cells and MAIT_cells (**Figure 7E**).

**Figure 7:**
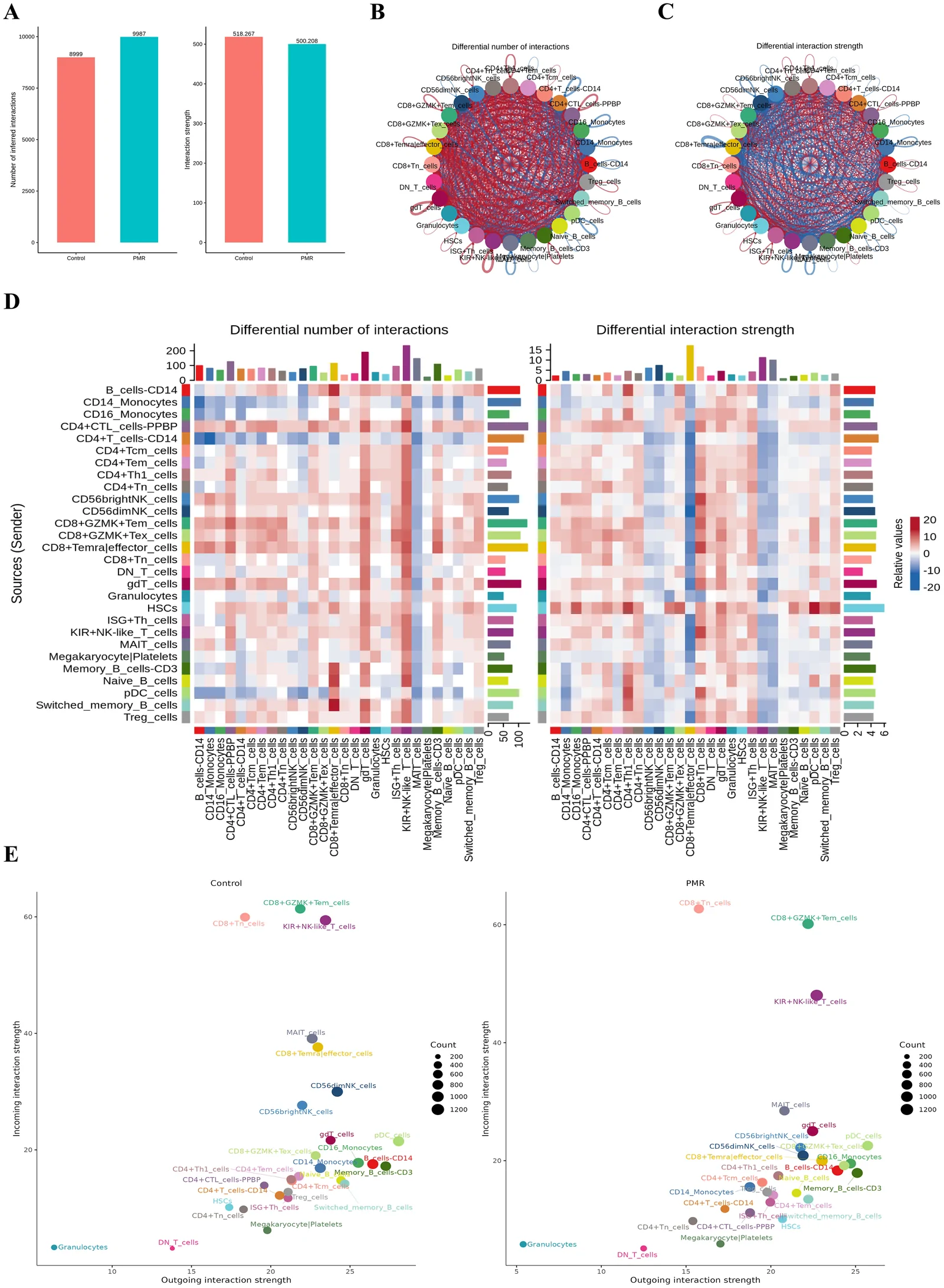
Analysis of the strength of communication between controls and PMRs at the single-cell level in PBMCs. **(A)** Number of interactions and interaction strength in controls and PMRs. **(B)** Differential number of interactions between controls and PMRs in each cell subtype. **(C)** Differential interaction strength between controls and PMRs in each cell subtype. **(D)** Heatmap of differential number of interactions and interaction strength between controls and PMRs in each cell subtype. **(E)** Identification of cell populations with significant variation in the outgoing or incoming signals in PBMCs.

Next, we identified conserved and environment-specific pathways by comparing the information flow of each pathway enriched by cell population interactions. By comparing the information flow/interaction strength of each signaling pathway, we could identify altered signaling pathways under different conditions. Our results showed the 9 distinct signaling pathways in controls (RESISTIN, TNF, NECTIN, CD96, TRAIL, TIGIT, SEMA3, CD226 and SEMA7) and 6 distinct signaling pathways in PMRs (IL1, CD39, FASLG, LIGHT, CCL and TGF-β). The overlapping communication pathways were COLLAGEN, CD99, ADGRE5, MHC-1, MIF, THBS, CLEC, LCK, CD34 (relative increase in controls), MHC-II, CD45, CD22, CD23, MPZ, CD86, CD6, ALCAM, IL16, GALECTIN, SEMA4, CXCL, PARs, PECAM1, ESAM, SELPLG, ICAM, CD40, ITGB2, CD48, ANNEXIN, BTLA and NCAM (relative increase in PMRs). The unique signaling pathways in PMRs attracted our attention (**Figures 8A, B**). For IL1 pathway, HSCs sent signals to MAIT_cells in PMR group and IL18-(IL18R1+IL18RAP) pairs were considered main contributors. For CD39 pathway, CD14_Monocyte and B_cells-CD14 sent signals to CD8^+^GZMK^+^Tex_cells in PMR group and ENTPD1-ADORA2A pairs were considered main contributors. For FASLG pathway (FASL-FAS pairs), CD56^bright^NK_cells, CD56^dim^NK_cells and KIR^+^NK-like T_cells were main senders, while CD4^+^Tcm_cells, CD4^+^Tem_cells, CD4^+^Th1_cells, CD4^+^T_cells-CD14, ISG^+^Th_cells and CD8^+^GZMK^+^Tex_cells, especially Treg_cells, were main receivers. For LIGHT pathway, CD56^bright^NK_cells, CD8^+^Temra/effector_cells, γδT_cells and MAIT_cells were main senders, while CD16_Monocytes and CD8^+^GZMK+Tex_cells were main receivers, although most cells expressed TNFRSF14. In this signaling pathway, TNFSF14-TNFRSF14 pairs were considered main contributors. In CCL pathway, most cells, especially CD56^bright^NK_cells, CD56^dim^NK_cells, CD8^+^GZMK^+^Tem_cells, CD8^+^Temra/effector_cells, γδT_cells, KIR^+^NK-like T_cells and megakaryocyte/platelets, were senders, and receivers were restricted in B_cells-CD14, CD14_Monocytes, CD4^+^Tem_cells and Treg_cells. In this signaling pathway, CCL5-CCR4 and CCL5-CCR1 pairs were considered main contributors. In terms of TGF-β pathway, most cells were senders, especially megakaryocyte/platelets, while CD56^bright^NK_cells, CD56^dim^NK_cells, CD8^+^GZMK^+^Tem_cells, CD8^+^Temra/effector_cells, γδT_cells and KIR^+^NK-like T_cells were main receivers. In this pathway, TGFB1-(TGFBR1+TGFBR2) pairs were main contributors (**Figure 8C**).

**Figure 8:**
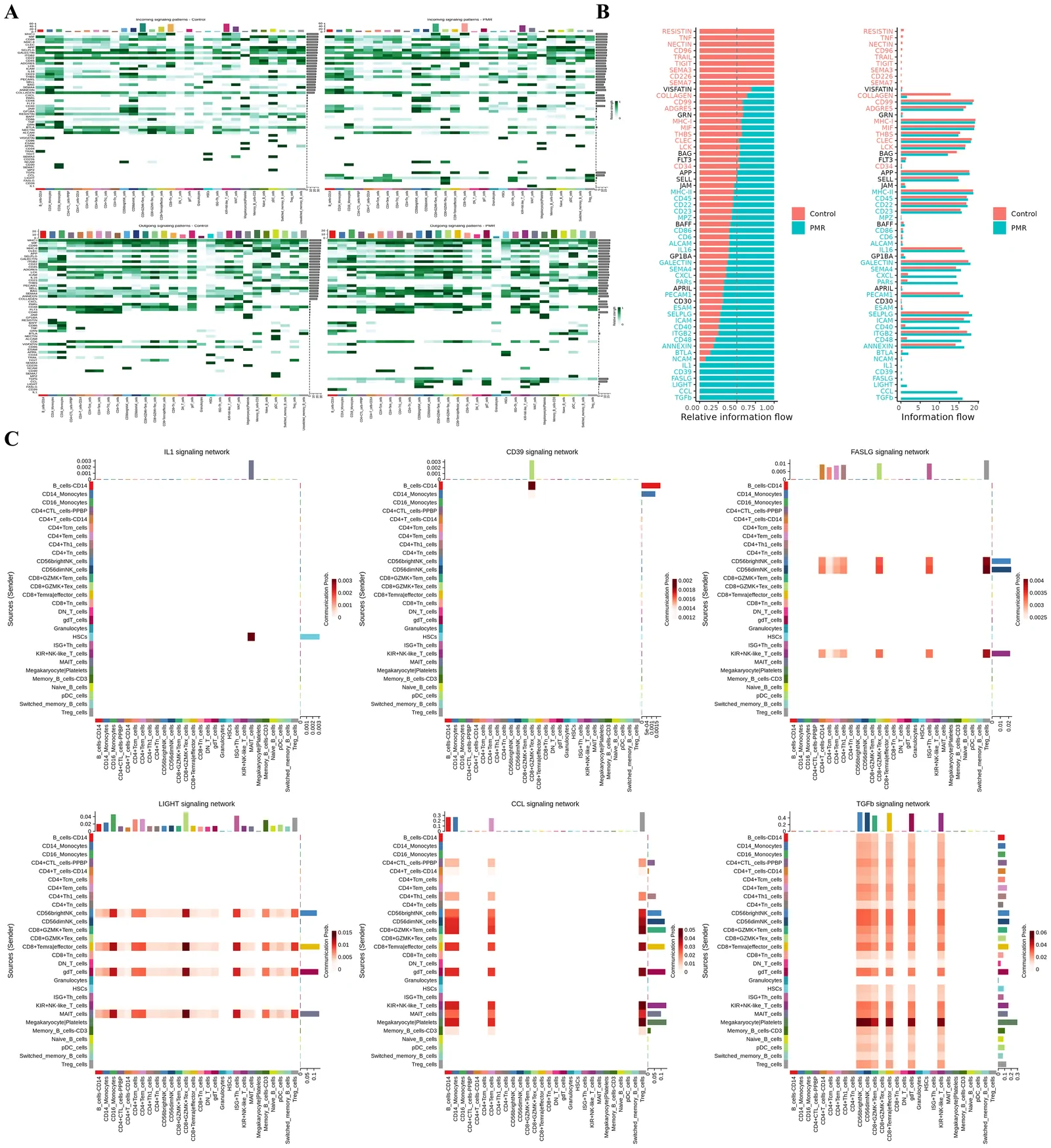
Analysis of the communication flow in controls and PMRs at the single-cell level in PBMCs. **(A)** Incoming and outgoing signal patterns of controls and PMRs. **(B)** Communication information flow in controls and PMRs. **(C)** Identification of the signaling roles of IL1 pathway, CD39 pathway, FASLG pathway, LIGHT pathway, CCL pathway and TGFb pathway in the cells.

We further selected IL1, CD39, FASLG, LIGHT, CCL, TGF-β, CXCL, PARs, CD40 and CD48 pathways for ligand-receptor pair analysis. Bubble plots show a number of ligand-receptor pairs with significant differences. For CD4^+^Tn_cells, CD4^+^Tcm_cells, CD4^+^Tem_cells, CD4^+^Th1_cells and ISG^+^Th_cells, they are mainly mediated by TGFB1-(TGFBR1+TGFBR2), CD48-CD244A, and CD40LG-(ITGAM+ITGB2) receptor-ligand pairs to act on other cells. For CD4^+^CTL_cells-PPBP, in addition to the three receptor-ligands mentioned above, PF4V1-CXCR3 and PF4-CXCR3 were also involved in the interactions of CD4^+^CTL_cells-PPBP with other cells (**Supplementary Figure S2**). For CD8^+^GZMK^+^Tem_cells, CD8^+^GZMK^+^Tex_cells, CD8^+^Temra/effector_cells, KIR^+^NK-like T_cells, CD4^+^T_cells-CD14, MAIT_cells and γδT_cells, they rely mainly on TGFB1-(TGFBR1+TGFBR2), CD48-CD244A and GZMA-F2R receptor-ligand pairs acting on other cells (**Supplementary Figures S3** and **S4**). For CD8^+^Tn_cells, DN T_cells, Treg_cells, CD14^+^ monocytes and CD16^+^ monocytes, they relied mainly on TGFB1-(TGFBR1+TGFBR2) and CD48-CD244A receptor-ligand pairs acting on other cells (**Supplementary Figures S3** and **S4**). Overall, B cell subsets acted primarily through TGFB1-(TGFBR1+TGFBR2) and CD48-CD244A receptor-ligand pair. B_cells-CD14, Memory_B_cells-CD3, Naive_B_cells and switched memory_B_cells acted mainly through TGFB1-(TGFBR1+TGFBR2) on CD56^bright^NK_cells, CD56^dim^NK_cells, CD8^+^GZMK^+^Tem_cells, CD8^+^Temra/effector_cells, γδT_cells and KIR^+^NK-like T_cells in PMRs (**Supplementary Figure S5**). Meanwhile, they also acted on CD16_Monocytes, CD56b^right^NK_cells, CD56^dim^NK_cells, γδT_cells and KIR^+^NK-like T_cells via CD48-CD244A, and the signaling intensity of the PMR group was higher than that of the control group (**Supplementary Figure S5**). NK cells acted on other cells through TGFB1-(TGFBR1+TGFBR2) and GZMA-F2R receptor-ligand pairs. HSCs and pDC_cells were associated with other cells through TGFB1-(TGFBR1+TGFBR2). Megakaryocyte/platelets were associated with other cells through PF4V1-CXCR3 and PF4-CXCR3 (**Supplementary Figure S6**).

## 4 Discussion

A better understanding of the complex interactive regulatory network between the innate and adaptive immune systems in PMR is critical to unraveling the pathogenesis of this disease and identifying effective treatments. In our study, we conducted a comprehensive exploration of the single-cell transcriptome and cellular communication in PMR patients and controls. Through our analyses, we identified 10 major cell types and highlighted distinct transcriptional heterogeneity within their cellular subpopulations. PMR patients exhibit profound innate and adaptive immune dysregulation at the single-cell level. These preliminary single-cell-level findings systematically delineate the co-activation landscape of both innate and adaptive immunity in PMR, an immune-mediated inflammatory disease, offering mechanistic insights into the complex dysregulated immune processes underlying its pathogenesis and may providing a foundation for future research and the development of targeted therapeutic interventions.

In our study, we first analyzed PBMCs as a whole and found a decrease in the proportion of total T cells. Clinically, PMR and giant cell arteritis (GCA) frequently present as comorbid conditions, and PMR can progress to overt GCA in a proportion of patients. Previous studies have reported a reduction in both the frequency and absolute count of circulating effector-memory CD8^+^ T cells in patients with GCA, with CD8^+^ T cells detected in temporal artery biopsies and aortic tissues of affected individuals (14). These findings led to the hypothesis that the decline in circulating effector-memory CD8^+^ T cells were attributable to tissue recruitment rather than global depletion (14). Consistently, synovitis in PMR has been characterized by infiltration of macrophages and T lymphocytes with high expression of HLA class II antigens (15). Based on these observations, we hypothesize that the reduction of circulating T cells in PMR is primarily driven by the recruitment and migration of T cells to sites of local inflammation, a mechanism shared across many autoimmune diseases associated with reduced peripheral blood lymphocytes. Meanwhile, similar to other autoimmune diseases, T cells reduction in PMR may also be the result of a combination of abnormal activation of the immune system, defective Treg function, chronic antigenic stimulation and metabolic reprogramming. In autoimmune diseases, over-activation of T cells (especially CD4^+^ T cells) leads to sustained depletion, along with a decrease in the number of Treg cells and abnormal function (16). Also in autoimmune diseases, prolonged exposure to self-antigens leads to continued activation of T cells, which gradually enter a state of exhaustion. Exhausted T cells exhibit poor proliferative potential; high inhibitory receptor expression; altered transcriptome, epigenome, and metabolism; and, most importantly, reduced effector function (17). Moreover, in autoimmune diseases, T cells undergo metabolic reprogramming that may directly impair T cell function and viability (18). Platelets, on the other hand, underwent significant amplification, which is consistent with the clinical features of PMR patients. In addition, there was a trend toward elevated CD14^+^ in the PMR group, suggesting enhanced antigen processing and presentation during PMR pathogenesis.

As major players in the inflammatory process, T cells play a key role in the pathogenesis of PMR. Although the proportion of total T cells is reduced, their function is significantly changed. T cell dysfunction has been reported in patients with PMRs. Patients with PMR have a significant increase in Th17 cells and a decrease in Th1 and Treg cells in their peripheral blood (19), and glucocorticosteroid treatment corrects the imbalance between Th17 cells and Treg cells (19). Despite similar concentrations of peripheral Th17 progenitor cells (CD161^+^CD4^+^T cells) in PMR and controls, *in vitro* IL-17 production was higher in PMR patients (19). Although no Th17 cells subpopulation was isolated in our study, on KEGG analysis of DEGs from T cells in the PMRs and controls, we found a significant enrichment of the Th17 cell differentiation pathway. Our study, however, found no evidence of Treg cells reduction, which may be related to the small sample size. In addition, we found that the proportions of CD4^+^Tcm cells, CD4^+^Tem cells and Th1 cells were reduced. The reduced proportion of Th1 cells may be related to their migration from the peripheral blood to the tissues, as previous studies have found that in synovial fluid and bursal tissue, an increase in Th1 cells rather than Th17 cells and an increase in IFN-γ rather than IL-17 in PMR patients (20). For the reduction of CD4^+^ Tcm cells and CD4^+^ Tem cells, we speculate that it may be related to the abnormal T cells differentiation. In the PMR autoimmune microenvironment, pro-inflammatory signals overactivate effector T cells subsets such as Th1 and Th17, whereas suppressive signals under-induce regulatory Treg cells, disrupting the Th17/Treg balance. This differentiation bias makes T cells more inclined to differentiate toward effector or exhausted phenotypes rather than memory T cells. The exhausted T cells are firstly unable to differentiate effectively into memory T cells and secondly also highly express immune checkpoint molecules, which inhibit the activation of self-reactive T cells, and thus hinder the formation of memory T cells (16, 17). Elevated levels of soluble co-inhibitory immune checkpoint molecules (particularly PD-1, PD-L1 and PD-L2) in patients with PMR and GCA, as compared with healthy controls, further support this assumption (21). More importantly, we also found that the proportion of KIR^+^NKT cells was higher in the PMR group. This group of NKT cells highly expressed KIR family and KLR family in addition to cytotoxic genes. In terms of functional enrichment, these cells had natural killer cell-mediated immunity or cytotoxicity. KIR^+^NKT cells in the PMR group scored higher than controls in antigen processing and presentation, inflammation promotion, cytotoxicity, and co-stimulation, suggesting that these cells may play an important role in the pathogenesis of PMR.

Our present study found a reduced percentage of peripheral blood B cells in the PMR group. This is consistent with previous studies, which have shown that the reduction in the number of circulating B cells during disease activity is associated with the redistribution of effector B cells (22, 23). The number and distribution of B cells in the peripheral blood of PMR patients treated with glucocorticoids and tolizumab (monoclonal antibody to the IL-6 receptor) were corrected (24). However, although B cells are reduced in circulating blood, they are rarely found in the synovial fluid of patients with PMR (20).

NK cells play an important role in the human immune system; they have the ability to directly kill target cells and are involved in regulating the immune response. Previous studies have documented a significant reduction in the absolute number of circulating NK cells in patients with PMR (25). This observation, along with similar findings in GCA (26), suggests a skewing of leukocyte distribution toward the myeloid lineage, which may alternatively reflect the migration of leukocytes from peripheral blood into inflamed tissues. NK cells can be classified into two different subtypes based on the expression of their surface markers. CD56^dim^CD16^hi^ NK cells express a variety of cytotoxic receptors, such as KIR and IL2R, with high cytotoxicity (27, 28). CD56^bright^CD16^low^ NK cells produce numerous cytokines and mainly play an immunomodulatory role (27, 28). In our study, CD56^bright^CD16^hi^ NK cells displayed intermediate features between CD56^bright^CD16^low^ and CD56^dim^CD16^hi^ NK cells, potentially representing developmental intermediates (29). Further, CD56^dim^CD16^hi^-PTGDS and CD56^dim^CD16^hi^-PPBP were found to be more prevalent in PMR patients. In particular, CD56^dim^CD16^hi^-ISG was present only in PMR patients. The CD56^dim^CD16^hi^-PTGDS cells, in addition to high expression of PTGDS, also express the chemokine receptors (CX3CR1 and CXCR2), the cytotoxic molecules (GZMH, GZMA, GZMB and PRF1), as well as the inflammatory molecules (CCL4L2, CCL5 and CCL4). As far as is known, CX3CR1-CX3CL1 and CCL4L2/CCL5/CCL4-CCR5 signaling pathways exert distinct functions in different tissue compartments, such as immune response, inflammation, cell adhesion and chemotaxis (30, 31). Thus, these signaling pathways may be responsible for the recruitment of NK cells to inflamed tissues. CD56^dim^CD16^hi^-PPBP cells expressed PPBP and PF4 and were elevated in PMRs, possibly representing the presence of cellular communication between CD56^dim^CD16^hi^ cells and platelets and possibly related to platelet activation. CD56^dim^CD16^hi^-ISG expressed interferon-stimulated genes, indicating that the interferon signaling pathway was involved in the pathogenesis of PMR.

Monocytes play a key role in autoimmune diseases, and changes in their number and function directly affect disease severity and progression. Based on different markers, monocytes are classified as CD14^+^ monocytes (CD14), CD16^+^ monocytes (FCGR3A) (32). Furthermore, we found the proportions of CD14_Monocyte-ISG cells, CD16_Monocyte-IFITM1 cells and CD16_Monocyte-NCF1 cells were higher in patients with PMR, while the proportions of CD16_Monocyte-NFKBIA cells were reduced. Both CD14_Monocyte-ISG cells and CD16_Monocyte-IFITM1 cells express ISGs. The increased proportions of these two cell types further suggest that interferon signaling pathway is activated in monocytes and is involved in the pathogenesis of PMR. CD16_Monocyte-NCF1 cells express genes associated with the intrinsic immune response and the processing and presentation of exogenous antigens, representing a class of cells that were involved in the above processes and thus in the pathogenesis of PMR. CD16_Monocyte-NFKBIA cells mainly expressed genes related to cellular stress response. Although the reason for the reduction of such cells in peripheral blood is unknown, we speculate that it may be related to their transfer to tissues, because the proportion of CD14_Monocyte-NFKBIA cells was also reduced in the PMRs in this study. Moreover, the presence of IL-6-expressing macrophages in the subacromial-subdeltoid bursa of patients with PMR has been previously reported in the literature (33).

Platelets, the smallest anucleate cell fragments in circulation derived from bone marrow megakaryocytes, exert multiple critical physiological functions including hemostasis, vascular wall integrity maintenance, immune-inflammatory regulation, and tissue repair. Our study observed a significant expansion of the platelet cluster in PMR, which aligns with the clinical feature of this disease. Accumulating evidence has implicated platelets in the pathogenesis of inflammatory arthritis and multiple autoimmune diseases including RA, systemic lupus erythematosus (SLE) and primary Sjögren’s syndrome (pSS) (34). Therefore, despite accounting for only 2.4% of total cells in our dataset, platelets may play an indispensable role in PMR pathogenesis. Further analysis revealed that megakaryocytes/platelets communicate with other cells primarily via PF4V1-CXCR3 and PF4-CXCR3 axes. Platelet signature genes PF4 and PPBP mediate thrombosis, inflammation and chemotaxis: PF4 (CXCL4) is a validated severity biomarker for Systemic sclerosis (SSc) and SLE (35), while PPBP (CXCL7) is closely linked to inflammation and serves as a potential indicator of elevated inflammatory activity across multiple diseases (36, 37).

It is worth highlighting that in the current study, we identified several atypical immune subpopulations with concurrent expression of canonical markers from two distinct immune lineages, including CD4^+^T_cells-CD14, Memory_B_cells-CD3, B_cells-CD14, CD56^dim^CD16^hi^-CD3, CD14_Monocyte-CD3, CD14_Monocyte-CD8 and CD16_Monocyte-CD3. These rare ambiguous populations were not directly excluded at this primary stage, as standard single-cell sequencing quality control and transcriptional profiling alone cannot definitively distinguish really dual-expressing subsets from heterotypic cell-cell complexes generated during experimental processing. The clear classification of these populations will require validation via independent experimental approaches, such as flow cytometry and imaging flow cytometry. This strategy also avoids predefined quality control thresholds prematurely eliminating real rare immune subsets, supports a complete, unbiased cellular composition landscape of this cohort, and preserves traceable raw data for subsequent validation and mechanistic studies on these underreported atypical immune populations.

Importantly, growing evidence across multiple autoimmune diseases confirms that dual-expressing cells are not technical artifacts, but functionally relevant players in the pathogenesis of systemic and organ-specific autoimmunity. Disease-associated dual-expressing subsets have been documented: a disease-linked CD19^+^CD14^+^ atypical B cell subset in active SLE (38), TCR/BCR co-expressing lymphocytes as key autoimmunity drivers in Type 1 diabetes (39), myelin-reactive TCR/IgM dual-expressing lymphocytes (40) and CD8^+^CD20^+^ dual-expressing T cells in multiple sclerosis (41), and a recently reported tissue-resident LPS-modulated CD14^+^CD8^+^ T cell subset (42). Together, these consistent, corroborating observations form a robust evidence chain, strongly supporting that dual-expressing cells are a previously underappreciated shared pathogenic immune module across the autoimmune disease spectrum, rather than isolated study-specific artifacts. Besides, we also identified several non-platelet cell subtypes expressing PPBP and PF4, including CD4^+^CTLs, CD56^dim^CD16^hi^ and CD14^+^/CD16^+^ monocytes. Platelet-signature scoring (43) showed that the scores of these cells were significantly lower than platelets but slightly higher than other cell subtypes (**Supplementary Table S7** (43) and **Supplementary Figure S7**), indicating that PP4 and PPBP expression is likely intrinsic to these subpopulations rather than derived from platelet contamination. Two hypotheses may explain this expression pattern: 1) These cells retain their original identity while acquiring a platelet-related transcriptional profile, which reflects inflammatory environment-induced transcriptional reprogramming rather than genuine lineage trans-differentiation (44); 2) This pattern reflects intercellular crosstalk, where platelet-derived extracellular vesicles (pEVs) deliver PF4/PPBP mRNAs from activated megakaryocytes/platelets to immune cells under inflammatory conditions, resulting in detectable signals (45). Both hypotheses require further experimental validation. Consistently, similar PF4^+^/PPBP^+^ megakaryocyte-like granulocytes (44) and PF4^+^/PPBP^+^ monocytes (46) with immunomodulatory functions have been reported in other autoimmune and inflammatory disorders.

Functional enrichment analyses revealed immune activation in PMRs when compared with controls. Pathways like natural killer cell mediated cytotoxicity, chemokine signaling pathway, NOD-like receptor signaling pathway and platelet activation were activated in most of the cell types. And that helps explain why platelets are increased in PMR. Also, KEGG analysis revealed evidence of pathogen infection, as previous studies described (2, 3). Although the pathogenesis of PMR is still unclear, most scholars believe that infection is one of the triggers of PMR pathogenesis. Possible mechanisms by which infections are associated with autoimmune diseases include target cells alterations, immune cells alterations, immune responses against viral and host shared determinants and cross-reactivity between specific and antiviral antibodies and autoantigens (47). It has been suggested that pathogens such as Mycoplasma pneumoniae, S. cerevisiae B19, adenoviruses, and respiratory syncytial viruses may induce PMR by inducing activation of monocytes/dendritic cells and production of pro-inflammatory cytokines (48). The GSVA results provided more information for understanding the pathophysiology of PMR. For the PBMCs in PMRs group, the common activated pathways included apical junction, complement, interferon alpha response, interferon gamma response and glycolysis. The above findings suggest a role for the immune response, particularly the interferon signaling pathway, in PMRs. Meanwhile, our study found that the proportions of NK cells and CD14^+^/CD16^+^ monocytes expressing ISGs were also upregulated in patients with PMR, which again emphasizes that type I interferon (IFN-I) may play an important role in PMR. The fact that interferon itself has antiviral and immunomodulatory effects side by side suggests that viral infection is involved in the pathogenesis of PMR. In other studies, the interferon pathway has also been found to be activated in PMR patients (49, 50). However, one study has noted that the IFN-I signature cannot sensitively discriminate GCA/PMR patients from healthy controls (51). This inconsistency may arise because they analyzed only bulk PBMCs without cell subtype stratification, and used low-resolution methods like PCR and serum immunoassays, which cannot reach the single-cell resolution of scRNA-seq. Notably, other studies have found that IFN-I-driven immune activation occurs before subclinical joint inflammation in specific immune subsets (e.g., circulating CD14^+^ classical monocytes and GZMK^+^CD8^+^ T cells), further supporting the critical role of IFN-I-mediated innate and adaptive immunity in autoimmune disease pathogenesis (52). Given that several biologic therapies that target the interferon system are in the pipeline, interferon activation in PMRs may be critical for future clinical trials, for example, those with high levels of interferon activation may benefit more from interferon blockade. In the resting state, immune cells such as lymphocytes rely primarily on catabolic reactions such as fatty acid oxidation and oxidative phosphorylation to meet their energy requirements (53, 54). Upon activation, metabolic conversions may be required to support the differentiation or enhancement responses of these cells (53, 54). The present study found that the activation of glycolysis metabolism occurs mainly in monocytes, suggesting that monocytes complete the metabolic switch to accommodate their immune response. In addition, enhanced fatty acid metabolism was found in B cells and T cells of PMR.

The complex network between innate and adaptive immune system in PMR is supported by the analysis of cellular communication. We first found an increase in outgoing messages from KIR^+^NK-like T_cells, and, in addition, a significant increase in the number of KIR^+^NK-like T_cells acting as receptor cells and communicating with most ligand cells. The above suggests that KIR^+^NK-like T_cells may be active players in the pathogenesis of PMR. We next identified 6 unique signaling pathways (IL1, CD39, FASLG, LIGHT, CCL and TGFb) in the PMR. Notably, in this study, we found that CD56^bright^NK_cells, CD56^dim^NK_cells, and KIR^+^NK-like T_cells could highly express FASL and bind to FAS expressed by CD4^+^Tcm_cells, CD4^+^Tem_cells, CD4^+^Th1_cells, CD4^+^T_ cells-CD14, ISG^+^Th_cells and CD8^+^GZMK+Tex_cells, especially Treg_cells, and induce their apoptosis. This may be another reason for the reduced ratios of CD4^+^Tcm_cells, CD4^+^Tem_cells and CD4^+^Th1_cells in this study. Furthermore, by observing ligand-receptor-mediated cell-cell communication, we found that the TGFb signaling pathway and its ligand-receptor pairs TGFB1-(TGFBR1+TGFBR2) should play an important role in PMR. In our study, almost all cells expressed TGF-β1, which acts in conjunction with the receptors TGFβR1 or TGFβR2 expressed on other cells (NK cells, CD8^+^ T cells, γδT cells and NKT cells). As a member of the TGF-β superfamily, TGF-β1 is the dominant isoform in immune cells and has pleiotropic immunosuppressive effects, including inhibition of T-cell activation, B-cell survival, proliferation, immunoglobulin synthesis, and promotion of peripheral Treg and Th17 cells differentiation (55). In patients with autoimmune diseases such as SLE, SSc, RA, pSS, Crohn’s disease, ulcerative colitis (UC), autoimmune hepatitis (AIH), and Hashimoto’s thyroiditis (HT), the levels of TGF-β or TGFβR in the tissues or circulation correlate positively or negatively with the presence, activity, and severity of the disease (56). Some cases of the diseases are likely caused by insufficient TGF-β expression and thus exhibit decreased TGF-β production (57). In some cases, autoimmune inflammation may be due to impaired cellular responsiveness to TGF-β, particularly due to defective TGFβR function, so that TGF-β production is elevated as a compensatory response (58). Therefore, we speculate that activation of the TGF-β signaling pathway in PMRs may fall into the latter category, being a compensatory elevation of the activated immune response. In addition, the CD48 signaling pathway and its ligand-receptor pair CD48-CD244 are another class of pathways that may play an important role in the pathogenesis of PMR. In our study, all T cells, B cells and monocytes express CD48, which communicates with other cells by binding to CD244. CD48 belongs to the signaling lymphocyte activation molecule family receptor family (SLAMF) along with CD244. CD48 is expressed on almost all hematopoietic cells (59) and can be increased especially under the influence of inflammatory factors. CD244 is a type 1 transmembrane protein that is present in NK cells, T cells and other types of immune cells (60). CD244 binds to CD48 on immune cells and regulates the immune response mainly through trans interactions (61), and the functional outcome of CD48-CD244 binding may vary in different cells. CD48-CD244 could affect the onset of autoimmune diseases by regulating various functions of immune cells and by maintaining or altering the balance of immune responses (60). Therefore, CD48-CD244, as a key effector molecule of immune response, is likely to be an important biomarker or therapeutic target for PMR, and further studies are needed in the future. Finally, we found that CD4^+^CTL_cells-PPBP and megakaryocyte/platelets also communicate with other cells via the PF4V1-CXCR3 and PF4-CXCR3 ligand receptor pairs, which may reflect active cellular communication.

In conclusion, this preliminary exploratory study represents the first transcriptome sequencing analysis of PBMCs from PMR patients. We hope these findings will contribute to a better understanding of PMR pathogenesis, and provide a useful reference for the identification of diagnostic markers and the development of novel therapeutic targets in future research. However, this study has some limitations. First, the sample size of this study is small, and the cases and controls are from different platforms. Although we mitigated batch effects via dedicated algorithms during data integration, confounding factors including age, gender, race and genetic background could influence immune cell composition, state and function, which remains a limitation of the current exploratory study. Further validation in larger independent cohorts is required to confirm our findings. Second, lack of crucial experimental validation. In future studies, we will perform further experimental validation at the protein level, including verifying the expression, function and mechanistic roles of key genes, as well as confirming the identified cell subtypes. Finally, our study currently does not address the potential association between PMR and GCA. Future single-cell level analysis will allow us to preliminarily explore the comorbid relationship between these two autoimmune rheumatic diseases.

## Data Availability

The data reported in this paper have been deposited in the OMIX, China National Center for Bioinformation / Beijing Institute of Genomics, Chinese Academy of Sciences (https://ngdc.cncb.ac.cn/omix: accession no.OMIX021840).

## Supplementary material

Supplementary Figure 1 Functional enrichment analysis. **(A)** GSVA plot shows the difference in pathway activity scores in PBMCs, T cells, B cells, NK cells, CD14^+^ monocytes and CD16^+^ monocytes between PMRs and controls. **(B)** Immunoreactivity scoring of subgroups of PBMCs between PMRs and controls. **(C)** Immunoreactivity scoring of subgroups of T cells between PMRs and controls. **(D)** Immunoreactivity scoring of subgroups of B cells between PMRs and controls. **(E)** Immunoreactivity scoring of subgroups of NK cells between PMRs and controls. **(F)** Immunoreactivity scoring of subgroups of CD14^+^ monocytes between PMRs and controls. **(G)** Immunoreactivity scoring of subgroups of CD16^+^ monocytes between PMRs and controls.

Supplementary Figure 2 Bubble plots of ligand-receptor interactions of CD4^+^T cell subsets with other cells between PMRs and controls (including IL1, CD39, FASLG, LIGHT, CCL, TGF-β, CXCL, PARs, CD40 and CD48 pathways). **(A)** CD4^+^Tn_cells with other cells. **(B)** CD4^+^Tcm_cells with other cells. **(C)** CD4^+^Tem_cells with other cells. **(D)** CD4^+^Th1_cells with other cells. **(E)** CD4^+^CTL_cells-PPBP with other cells. **(F)** ISG^+^Th_cells with other cells.

Supplementary Figure 3 Bubble plots of ligand-receptor interactions of CD8^+^T cell subsets/KIR^+^NKT cells/CD4^+^T_cells-CD14 cells with other cells between PMRs and controls (including IL1, CD39, FASLG, LIGHT, CCL, TGF-β, CXCL, PARs, CD40 and CD48 pathways). **(A)** CD8^+^Tn_cells with other cells. **(B)** CD8^+^GZMK^+^Tem_cells with other cells. **(C)** CD8^+^GZMK^+^Tex_cells with other cells. **(D)** CD8^+^Temra/effector_cells with other cells. **(E)** KIR^+^NKT cells with other cells. **(F)** CD4^+^T_cells-CD14 with other cells.

Supplementary Figure 4 Bubble plots of ligand-receptor interactions of DN T_cells/γδT_cells/Treg_cells/MAIT_cells/CD14^+^monocytes/CD16^+^monocytes with other cells between PMRs and controls (including IL1, CD39, FASLG, LIGHT, CCL, TGF-β, CXCL, PARs, CD40 and CD48 pathways). **(A)** DN T_cells with other cells. **(B)** γδT_cells with other cells. **(C)** Treg_cells with other cells. **(D)** MAIT_cells with other cells. **(E)** CD14^+^monocytes with other cells. **(F)** CD16^+^monocytes with other cells.

Supplementary Figure 5 Bubble plots of ligand-receptor interactions of B cell subsets with other cells between PMRs and controls (including IL1, CD39, FASLG, LIGHT, CCL, TGF-β, CXCL, PARs, CD40 and CD48 pathways). **(A)** B_cells-CD14 with other cells. **(B)** Memory B cells-CD3 with other cells. **(C)** naive_B_cells with other cells. **(D)** switched memory B cells with other cells.

Supplementary Figure 6 Bubble plots of ligand-receptor interactions of NK cell subsets/HSCs/pDC/megakaryocyte/platelets with other cells between PMRs and controls (including IL1, CD39, FASLG, LIGHT, CCL, TGF-β, CXCL, PARs, CD40 and CD48 pathways). **(A)** CD56^bright^NK cells with other cells. **(B)** CD56^dim^NK cells with other cells. **(C)** HSCs with other cells. **(D)** pDC with other cells. **(E)** megakaryocyte/platelets with other cells.

Supplementary Figure 7 Platelet-signature scoring for the 50 cell subtypes in this study using the function “AddModuleScore” provided by Seurat Package. The platelet gene set was from the literature (43).
