# Supplementary material for: Single-cell transcriptome sequencing analysis of peripheral blood mononuclear cells reveals immune signatures in patients with polymyalgia rheumatica
Source: Front Immunol. 2026 Oct 19;17:1852877. doi: 10.3389/fimmu.2026.1852877 (PMC13724368; doi:10.3389/fimmu.2026.1852877)
Supplement: Supplementary file 1 [file Table1.docx]

Supplementary Table S1　Clinical characteristics of PMR patients and controls

| Sample  Indicators | PMR1 | PMR2 | PMR3 | Control1 | Control2 | Control3 |
| --- | --- | --- | --- | --- | --- | --- |
| Sex | female | female | female | female | female | female |
| Age | 58 | 67 | 60 |  |  |  |
| Age of onset | 58 | 67 | 60 |  |  |  |
| HGB (g/L) | 104 | 92 | 101 |  |  |  |
| Platelets (10^9/L) | 376 | 426 | 420 |  |  |  |
| ESR (mm/h) | 99.00 | 95.00 | 82.00 |  |  |  |
| CRP (mg/L) | 150.45 | 56.43 | 34.27 |  |  |  |
| C3 (g/L) | 1.64 | 1.20 | 1.30 |  |  |  |
| C4 (g/L) | 0.36 | 0.19 | 0.28 |  |  |  |
| IgA (g/L) | 4.47 | 2.98 | 4.02 |  |  |  |
| IgG (g/L) | 13.90 | 22.2 | 19.70 |  |  |  |
| IgM (g/L) | 0.75 | 1.11 | 0.68 |  |  |  |
| ASO (IU/mL) | <53.40 | 77 | 79 |  |  |  |
| RF (IU/mL) | <9.38 | <9.19 | <9.19 |  |  |  |
| Anti-CCP (U/mL) | 3.4 | 2.2 | <0.5 |  |  |  |
| Anti-dsDNA | negative | negative | negative |  |  |  |
| Anti-SSA | negative | negative | negative |  |  |  |
| Anti-SSB | negative | negative | negative |  |  |  |
| ANA | negative | negative | negative |  |  |  |
| TPOAb (U/mL) | 228.46 | 0.61 | 18.66 |  |  |  |
| TgAb (U/mL) | 7.85 | 1.77 | 8.99 |  |  |  |
| TSH (uIU/mL) | 2.709 | 0.52 | 1.640 |  |  |  |
| CK-MB (ng/mL) | <0.80 |  | <1.00 |  |  |  |
| CTnI (ng/mL) | <0.01 |  | <0.025 |  |  |  |
| MYO (ng/mL) | 9.0 |  | 6.7 |  |  |  |
| ALP (U/L) | 110 | 58 | 58 |  |  |  |
| ALT (U/L) | 12 | 17 | 17 |  |  |  |
| AST (U/L) | 15 | 18 | 20 |  |  |  |
| Datasets |  |  |  | 10k Human PBMCs, 5' v2.0, Chromium Controller | 10k Human PBMCs, 5' v2.0, Chromium X | 20k Human PBMCs, 5' HT v2.0 |

Reference range: HGB, 115~150 (g/L); Platelets, 100~400 (10^9/L); ESR, 0~20 (mm/h); CRP, 0~10 (mg/L); C3, 0.80~1.60 (g/L); C4, 0.16~0.47 (g/L); IgA, 0.90~4.50 (g/L); IgG, 6.94~16.20 (g/L); IgM, 0.60~2.80 (g/L); ASO, <200 (IU/mL); RF, 0~15.90 (IU/mL); Anti-CCP, <5.00 (U/mL); TPOAb, 0~5.61 (U/mL); TgAb, 0~4.11 (U/mL); TSH, 0.350~4.940 (uIU/mL); CK-MB, 0~5.85 (ng/mL); CTnI, 0~0.10 (ng/mL); MYO, 0~100 (ng/mL); ALP, 42~141 (U/L); ALT, 13~69 (U/L); AST, 15~46 (U/L)
