# Supplementary material for: Single-cell transcriptome sequencing analysis of peripheral blood mononuclear cells reveals immune signatures in patients with polymyalgia rheumatica
Source: Front Immunol. 2026 Oct 19;17:1852877. doi: 10.3389/fimmu.2026.1852877 (PMC13724368; doi:10.3389/fimmu.2026.1852877)
Supplement: Supplementary file 2 [file Table3.docx]

Supplementary Table S3　Summary of scRNA-seq data of PBMCs

| Sample | Cells | Median reads per cell | Median genes per cell | Total genes detected | Median UMI counts per cell | Number of cells passing QC |
| --- | --- | --- | --- | --- | --- | --- |
| Control1 | 9,083 | 23,237 | 1,697 | 23,210 | 4,825 | 7,560 |
| Control2 | 9,129 | 25,901 | 1,778 | 23,487 | 5,057 | 7,552 |
| Control3 | 17,581 | 29,692 | 1,870 | 24,157 | 5,705 | 15,174 |
| PMR1 | 13,066 | 18,505 | 1,619 | 22,998 | 3,869 | 12,079 |
| PMR2 | 9,437 | 26,165 | 1,933 | 23,044 | 4,801 | 8,591 |
| PMR3 | 9,702 | 25,963 | 1,820 | 22,622 | 3,856 | 8,408 |
