# Supplementary material for: Single-cell transcriptome sequencing analysis of peripheral blood mononuclear cells reveals immune signatures in patients with polymyalgia rheumatica
Source: Front Immunol. 2026 Oct 19;17:1852877. doi: 10.3389/fimmu.2026.1852877 (PMC13724368; doi:10.3389/fimmu.2026.1852877)
Supplement: Supplementary file 3 [file Table7.docx]

**Supplementary Table S7.** Platelets-related genes used in platelet-signature scoring

| Gene symbols | | | | | | | |
| --- | --- | --- | --- | --- | --- | --- | --- |
| A1BG | A2M | AAMP | ABCC4 | ABHD12 | ABHD6 | ABL1 | ACTB |
| ACTN1 | ACTN2 | ACTN4 | ADRA2A | ADRA2B | ADRA2C | AHSG | AK3 |
| AKAP1 | AKAP10 | AKT1 | ALB | ALDOA | ANXA5 | APBB1IP | APLP2 |
| APOA1 | APOH | APOOL | APP | ARRB1 | ARRB2 | ASAH1 | BCAR1 |
| BEX3 | BRPF3 | CABLES1 | CABLES2 | CALM1 | CALU | CAP1 | CAPZA1 |
| CAPZA2 | CAPZB | CARMIL1 | CBX5 | CCL5 | CD109 | CD36 | CD63 |
| CD9 | CDC37L1 | CDC42 | CDK2 | CDK5 | CENPE | CFD | CFL1 |
| CHID1 | CLEC1B | CLEC3B | CLU | COL1A1 | COL1A2 | CRK | CSK |
| CTSA | CTSW | CTTN | CXCL5 | CYB5R1 | CYRIB | DAGLA | DAGLB |
| DAPP1 | DGKA | DGKB | DGKD | DGKE | DGKG | DGKH | DGKI |
| DGKK | DGKQ | DGKZ | DOCK1 | DOCK10 | DOCK11 | DOCK2 | DOCK3 |
| DOCK4 | DOCK5 | DOCK6 | DOCK7 | DOCK8 | DOCK9 | ECM1 | EGF |
| EHD1 | EHD2 | EHD3 | EIF2AK1 | ENDOD1 | F13A1 | F2 | F2R |
| F2RL2 | F2RL3 | F5 | F8 | FAM3C | FCER1G | FERMT3 | FGA |
| FGB | FGG | FHL1 | FLNA | FN1 | FYN | GAS6 | GATA1 |
| GATA2 | GATA3 | GATA4 | GATA5 | GATA6 | GLA | GNA11 | GNA12 |
| GNA13 | GNA14 | GNA15 | GNAI1 | GNAI2 | GNAI3 | GNAQ | GNAS |
| GNAT3 | GNB1 | GNB2 | GNB3 | GNB4 | GNB5 | GNG10 | GNG11 |
| GNG12 | GNG13 | GNG2 | GNG3 | GNG4 | GNG5 | GNG7 | GNG8 |
| GNGT1 | GNGT2 | GP1BA | GP1BB | GP5 | GP6 | GP9 | GRB2 |
| GTPBP2 | H2AC6 | H2BC21 | H3-3A | H3-3B | H3C1 | H3C10 | H3C11 |
| H3C12 | H3C13 | H3C14 | H3C15 | H3C2 | H3C3 | H3C4 | H3C6 |
| H3C7 | H3C8 | HABP4 | HBA1 | HBB | HBD | HBE1 | HBG1 |
| HBG2 | HDAC1 | HDAC2 | HGF | HMG20B | HRG | HSPA5 | IFNA1 |
| IFNA10 | IFNA13 | IFNA14 | IFNA16 | IFNA17 | IFNA2 | IFNA21 | IFNA4 |
| IFNA5 | IFNA6 | IFNA7 | IFNA8 | IFNB1 | IGF1 | IGF2 | IRF1 |
| IRF2 | ISLR | ITGA2B | ITGB3 | ITGB5 | ITIH3 | ITIH4 | ITPK1 |
| ITPR1 | ITPR2 | ITPR3 | JAK2 | JMJD1C | KDM1A | KIF11 | KIF12 |
| KIF13B | KIF15 | KIF16B | KIF18A | KIF18B | KIF19 | KIF1A | KIF1B |
| KIF1C | KIF20A | KIF20B | KIF21A | KIF21B | KIF22 | KIF23 | KIF25 |
| KIF26A | KIF26B | KIF27 | KIF2A | KIF2B | KIF2C | KIF3A | KIF3B |
| KIF3C | KIF4A | KIF4B | KIF5A | KIF5B | KIF6 | KIF9 | KIFAP3 |
| KIFC1 | KIFC2 | KLC1 | KLC2 | KLC3 | KLC4 | KNG1 | LAMP2 |
| LAT | LCK | LCP2 | LEFTY2 | LEPROT | LGALS3BP | LHFPL2 | LY6G6F |
| LYN | MAFF | MAFG | MAFK | MAGED2 | MANF | MAP3K7CL | MAPK1 |
| MAPK14 | MAPK3 | MAX | MFAP3L | MFN1 | MFN2 | MGLL | MICAL1 |
| MLH3 | MMD | MMRN1 | MPIG6B | MPL | MPP1 | MYB | MYLK |
| NAP1L1 | NFE2 | NHLRC2 | NRGN | ODC1 | OLA1 | ORM1 | ORM2 |
| P2RY1 | P2RY12 | PCDH7 | PCYOX1L | PDGFA | PDGFB | PDLIM1 | PDPK1 |
| PDPN | PECAM1 | PF4 | PF4V1 | PFN1 | PGRMC1 | PHACTR2 | PHF21A |
| PIK3CA | PIK3CB | PIK3CG | PIK3R1 | PIK3R2 | PIK3R3 | PIK3R5 | PIK3R6 |
| PIP4K2A | PLA2G4A | PLCG2 | PLEK | PLG | PPBP | PPIA | PPM1A |
| PRDX6 | PRKACA | PRKACB | PRKACG | PRKAR1A | PRKAR1B | PRKAR2A | PRKAR2B |
| PRKCA | PRKCB | PRKCD | PRKCE | PRKCG | PRKCH | PRKCQ | PRKCZ |
| PROS1 | PRUNE1 | PSAP | PTGS1 | PTK2 | PTPN1 | PTPN11 | PTPN12 |
| PTPN6 | QSOX1 | RAB27B | RAB31 | RAB5A | RABGAP1L | RAC1 | RAC2 |
| RACGAP1 | RAD51B | RAD51C | RAF1 | RAP1A | RAP1B | RAPGEF3 | RAPGEF4 |
| RARRES2 | RASGRP1 | RASGRP2 | RBSN | RCOR1 | RGS10 | RHOA | RHOB |
| RHOG | RNF11 | RSU1 | RUFY1 | RYBP | SCCPDH | SCG3 | SELENOP |
| SELP | SERPINA1 | SERPINA3 | SERPINA4 | SERPINE1 | SERPINF2 | SERPING1 | SH2B1 |
| SH2B2 | SH2B3 | SHC1 | SIN3A | SNCA | SNN | SOD1 | SOS1 |
| SPARC | SPP2 | SRC | SRGN | STX4 | STXBP2 | STXBP3 | SYK |
| SYTL4 | TAGLN2 | TAX1BP3 | TBXA2R | TEX264 | TF | TGFB1 | TGFB2 |
| TGFB3 | THBS1 | THPO | TIMP1 | TIMP3 | TLN1 | TMEM140 | TMSB4X |
| TMX3 | TNFSF4 | TOR4A | TP53 | TPM1 | TRPC3 | TRPC6 | TRPC7 |
| TSC22D1 | TTN | TUBA1A | TUBA1B | TUBA1C | TUBA3C | TUBA3D | TUBA3E |
| TUBA4A | TUBA4B | TUBA8 | TUBAL3 | TUBB1 | TUBB2A | TUBB2B | TUBB3 |
| TUBB4A | TUBB4B | TUBB6 | TUBB8 | TUBB8B | VAV1 | VAV2 | VAV3 |
| VCL | VEGFA | VEGFB | VEGFC | VEGFD | VPS45 | VTI1B | VWF |
| WDR1 | WEE1 | WIPF1 | YPEL5 | YWHAZ | ZFPM1 | ZFPM2 | ZNF185 |

Note: Citing from: Wang, Yufang, Yang, Junjie, Xu, Xiaofang, Yu, Tao, Construction of a Platelet - Related Gene Signature for Predicting Prognosis and Drug Sensitivity in Colorectal Cancer, European Journal of Cancer Care, 2026, 6430579, 11 pages, 2026.:// httpsdoi.org/10.1155/ecc/6430579
